# Functional Metagenomics Insights Into the *Allium ampeloprasum* Rhizosphere Microbiome Under Different Fertilization Regimes

**DOI:** 10.1002/mbo3.70307

**Published:** 2026-06-10

**Authors:** Oluwaseun Emmanuel Shittu, Ben Jesuorsemwen Enagbonma, Olubukola Oluranti Babalola

**Affiliations:** ^1^ Food Security and Safety Focus Area, Faculty of Natural and Agricultural Sciences North‐West University Mmabatho South Africa; ^2^ Department of Life Sciences Imperial College London, Silwood Park Campus Ascot Berkshire UK

**Keywords:** agricultural sustainability, biofertilizer, food security, leek, shotgun sequencing

## Abstract

Fertilization practices shape the taxonomy, functional composition, and metabolic functions of the microbiome within the rhizosphere. Nonetheless, the impacts of various fertilization approaches on the functional composition of *Allium ampeloprasum* rhizosphere microbiomes remain underexplored. This study investigated how biofertilizers and chemical fertilizers impact the microbial functional categories of the *A. ampeloprasum* rhizosphere, hypothesizing that fertilization systems influence the metabolic profile. The genomic DNA was successfully extracted from the collected soil samples and processed via shotgun metagenomics sequencing. The application of biofertilizers enhanced the rhizosphere microbiome, revealing similar microbial orders across all plots, although plot G2 was uniquely enriched with those belonging to phyla Bacteroidota, Proteobacteria, actinobacteria, Myxococcota, and Verrucomicrobiota. Biofertilizers promoted a broader range of microbial functions, primarily at EggNOG level 1. Notably, the α diversity significantly differed (*p* < 0.05) among the soil samples. The functional diversity was linked to the soil physicochemical attributes, particularly the carbon and moisture contents, as illustrated by the RDA. Biofertilizer increases microbial diversity, underscoring the need to understand the rhizosphere microbiome to advance sustainable agricultural methods.

## Graphical abstract

Shotgun metagenomic sequencing was used to characterize the functional potential of the *Allium ampeloprasum* rhizosphere microbiome under chemical fertilizer (G1), biofertilizer (G2), and unfertilized (Bulk) soil (G3) management practices. This study revealed that different fertilization regimes altered microbial functional profiles. Specifically, biofertilizer in G2 enriched functions associated with nutrient cycling, stress tolerance, and plant growth‐promoting processes. In summary, this study demonstrates that fertilization strategy significantly influences rhizosphere microbial functions, highlighting its importance for sustainable soil management, improved crop productivity, and agriculture.

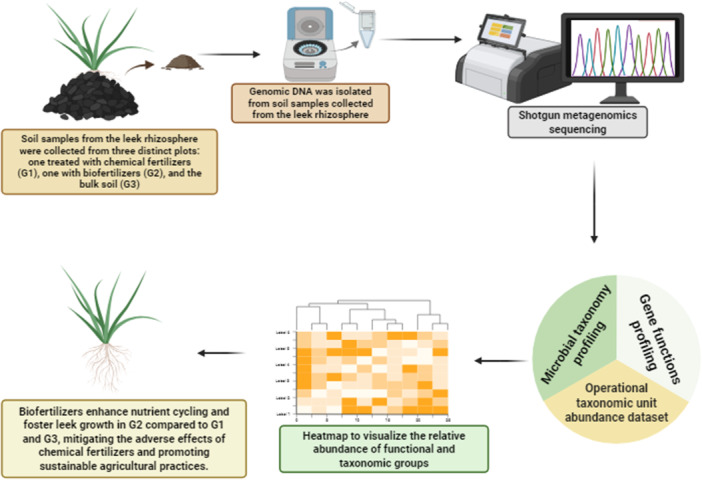

## 1 Introduction

Fertilization methods are fundamental to agricultural practices and significantly affect soil health, crop yield, and environmental sustainability (Sharma et al. 2024). However, their influence on the rhizosphere microbiome has not been thoroughly studied, especially in crops, such as *Allium ampeloprasum*. This vegetable crop is highly appreciated for its important culinary and health benefits. Packaging essential vitamins, minerals, and antioxidants is crucial for enhancing human nutrition and well‐being. Its versatility in terms of dishes and potential medicinal qualities makes it an important crop in global food production and traditional healing practices (Fakhfakh et al. 2024). Equally important is the microbiome that inhabits its rhizosphere. These microbiomes carry out pivotal functions in plant well‐being and development, facilitating nutrient cycling, increasing resistance to stress, and suppressing pathogens through their functional diversity (Figure S1) (Kapoor et al. 2024). These microbial diversities are further influenced by the production of secondary metabolites, such as phenolics, alkaloids, terpenoids, and flavonoids. These factors promote their colonization while suppressing harmful metabolites (Adedeji and Babalola 2020).

Characterizing the composition and functional diversity of rhizosphere microbiomes under various fertilization systems can offer valuable information for optimizing microbial activities. Such insights are vital for sustainable food production and align with Goal 2 of the United Nations' Developmental Sustainable goals, which seek to eliminate hunger by encouraging resilient farming practices (Jagadesh et al. 2024). In this context, Babalola et al. (2025) highlighted the role of biofertilizers in enriching microbial diversity and their functional potential (Figure S1). These effects are achieved by efficient root colonization and the promotion of beneficial microbial species. These include microorganisms that help solubilize insoluble phosphate and phytohormone production, activate plant immune responses, increase stress tolerance, and promote tomato fruit maturation (*Solanum lycopersicum*) Adedayo et al. (2023). However, the utilization of chemical fertilizers can alter the rhizosphere microbiome. They encourage microbes that thrive in nutrient‐rich environments with potassium, phosphorus, and nitrogen. While these fertilizers may increase short‐term plant development, they frequently reduce overall microbial diversity by suppressing nitrogen‐fixing bacteria, rhizobacteria, and mycorrhizal fungi (Kan et al. 2024). This decline can harm the process of nutrient cycling, deplete the organic matter of the soil, and weaken the natural defenses of plants against pathogens, ultimately lowering agricultural output and product quality (Zhang et al. 2023).

Moreover, the rhizosphere microbiome plays a central role in the cycling of nutrients by promoting diazotrophy (e.g., *Rhizobium* and *Azospirillum*), phosphorus solubilization (*Pseudomonas* and *Bacillus*), and iron chelation through siderophores. These processes improve nutrient availability while reducing the dependence on chemical fertilizers (Figure S1) (Babalola et al. 2021).

Shittu et al. (2025) characterized the rhizosphere microbiomes of *A. ampeloprasum* and identified members of the phyla Pseudomonadota, Myxococcota, Bacteroidota, and Verrucomicrobiota from a biofertilizer‐treated soil with plant growth‐promoting genes that contribute to *A. ampeloprasum* growth and development. Similarly, a study by Olanrewaju and Babalola (2019) further highlighted that bacterial consortia enhanced maize growth and development by facilitating the recruitment of beneficial microbes and their diverse functional potentials. Beneficial microbes also combat soil‐borne pathogens through antibiosis, competition, or plant systemic defense mechanisms (Khoso et al. 2024) while strengthening plant defenses against diverse living and nonliving stresses (Fadiji et al. 2023).

Given these crucial roles, profiling the microbial functional profile of the *A. ampeloprasum* rhizosphere under different fertilization practices is essential. Biofertilizer enhances nutrient absorption and crop vitality while minimizing reliance on conventional fertilizers, thus mitigating soil degradation and pollution. They can also increase the nutrient content of crops, such as *A. ampeloprasum*. Therefore, a comprehensive approach to fertilization strategies will not only strengthen localized agricultural systems but also contribute to food security worldwide (P. P. Das et al. 2022).

This approach directly supports Sustainable Development Goal (SDG) 2, especially Target 2.4, which emphasizes the need for sustainable farming methods to ensure the accessibility of food and enhance soil quality. Moreover, understanding microbial functions can guide precision agriculture and enhance resource efficiency while supporting SDG 2 goals (Kiprotich et al. 2025).

However, numerous microorganisms cannot be cultured via standard laboratory techniques, thereby limiting conventional studies. Shotgun sequencing overcomes this challenge by directly sequencing environmental DNA (Liu et al. 2022; Shittu et al. 2026). This method reveals microbial diversity, composition, richness, and functional diversity. This has deepened our understanding of microbial community dynamics and their contributions to agricultural sustainability (Nwachukwu and Babalola 2022).

Moreover, microbial communities perform different metabolic functions, including stress response, iron and aromatic compound metabolism, virulence, and defense (Enagbonma and Babalola 2023). Metagenomics has revolutionized the study of noncultivable microbes, thereby offering insights into their functional and taxonomic organization (Zhang and Zhang 2023). The shotgun metagenomics sequencing utilized in this investigation provides broader advantages than conventional microscopy and 16S ribosomal RNA (rRNA) sequencing by characterizing both taxonomic and functional diversity.

We harnessed a shotgun metagenomics sequencing technique in this study to profile the richness, makeup, and diversification of the microbial community functions of rhizosphere microbes associated with *A. ampeloprasum* under various fertilizer treatments in relation to uncultivated bulk soil. This sequencing technique has yielded valuable insights into the functional categories of microorganisms across crops, as well as between uncultivated and rhizosphere soils (Ajiboye et al. 2022; O. P. Omotayo et al. 2022). This strategy allowed us to adequately characterize the microbial functional characteristics across different soil types under different fertilization practices and in bulk soils.

This study therefore addresses three research questions: (1) How do fertilization systems impact the variety, structure, and richness of microbial functional diversity in the *A. ampeloprasum* rhizosphere? (2) Do the physiological parameters of soil shape the distribution of microbial functional categories? (3) Which metabolic pathways are most affected by fertilization practices, and what are the implications for soil health and development?

To the best of our knowledge, few studies have explored the microbial communities inhabiting the rhizosphere of *A. ampeloprasum*. This study presents one of the earliest works to comprehensively employ shotgun metagenomics sequencing to characterize the rhizosphere microbiomes linked to *A. ampeloprasum* across various fertilization systems in relation to its functional diversity.

## 2 Materials and Methodological Procedures

### 2.1 Experimental Site Profile

Rhizosphere soil samples were collected from Rosaly Farm (a commercial leek farm), which is located in Gauteng Province, a region within the sub‐Saharan portion of South Africa with geographic coordinates of 26°06′10.7″ S and 27°34′41.8″ E. This area is characterized by grassland ecosystems interspersed with shrubs and sparse tree plantations.

The climatic characteristics of this region are defined by an average temperature of 22°C, which reflects its conventional thermal conditions, encompassing both cooler and warmer periods. Annually, the region receives a precipitation level of approximately 794 mm, predominantly occurring between October and May, with pH levels characterized as mildly basic, varying between 7.21 and 7.33 (Table S1). Historically, this experimental agricultural site has been utilized for the cultivation of wheat and various vegetable species. Farm Plot A (G1) received chemical fertilizers, whereas Farm Plot B (G2) was treated with organic biofertilizers. Moreover, Farm Plot C (G3) did not receive any fertilizer.

Plot A has a documented history of mineral fertilizer application at the following rates per square meter (g m^−2^): Ca(NO_3_)_2_ at 150 g, (NH_4_)_2_SO_4_ at 120 g, KNO_3_ at 200 g, Mg(NO_3_)_2_) at 150 g, K_2_SO_4_ at 150 g, and N:P:K (a compound fertilizer in a proportion of 5:1:5) at 100 g. Moreover, plot B received applications per square meter (m^2^), Terramax (a naturally derived organic extract comprising a consortium of rhizobacteria that promote plant growth‐promoting rhizobacteria) at 0.56 g; Humesoil (a naturally derived organic blend composed of selected plant‐ and tree‐derived organic materials) at 0.82 L; and Soluphos (a microbial inoculant for phosphate solubilization in powdered organic form, composed of *Bacillus licheniformis* and *Pseudomonas putida*, applied at a concentration of 4.8 × 10^9^ CFU g^−1^) at 0.494 mL, together serving as microbial inoculants. Mineral fertilizers as well as organic amendments (biofertilizers) were applied to evaluate their effects on the structure and diversity of soil microbial communities, along with crop development, relative to untreated bulk soils, which served as the control (Table S8).

### 2.2 Soil Sampling Methods

The agricultural plot, from which the root‐associated soil and uncultivated soil samples were acquired, covered a total area of 15,000 m^2^. The farm plot was sectioned into three distinct sampling fields, each measuring 60 × 60 m, resulting in a total area of 3600 m^2^ for each plot. The first experimental field, designated G1, was subjected to treatment with inorganic fertilizers (chemical), and a biofertilizer treatment was applied to the second experimental field (G2), whereas the third farm plot (G3), identified as uncultivated bulk soil, remained unfertilized, with each plot spaced 20 m apart from the others to mitigate any potential interaction effects among treatments and to ensure adequate experimental separation.

The soils originating from the rhizosphere were carefully obtained from the roots of viable *A. ampeloprasum* plants, with a spatial separation of 45 m between each plant. However, the bulk soil is characterized as uncultivated land devoid of *A. ampeloprasum* cultivation. The rhizosphere soils were procured from the proximity of the *A. ampeloprasum* plant roots via a sterile soil auger. The instrument enabled the extraction of soil to a depth of 150 mm. The rhizosphere soil samples were subsequently secured following a gentle agitation process to dislodge the adhering bulk soil from the plant root, and the samples were then collected in sterile zip‐lock plastic bags for preservation. The soil samples were subsequently separated and sieved, followed by storage in airtight containers at a temperature of 4°C (Shittu et al. 2025).

Rhizosphere samples were procured from *A. ampeloprasum* plants 57 days after planting during their flowering (bolting) phase. Concurrently, bulk soil samples were collected from an adjacent uncultivated plot located 20 m from the treated plots to minimize the potential confounding impacts of fertilizers as well as root exudates.

For each fertilizer treatment (G1 and G2) and the bulk soil condition (G3), four rhizosphere soil cores were randomly collected per plot, yielding a total of 12 biological replicates (3 plots × 4 cores). A composite sample was generated by pooling and homogenizing four rhizosphere soil samples collected from each plot, and these composite samples were subsequently processed as independent units. This sampling strategy was designed to characterize the rhizosphere soil under the heterogeneous microenvironmental conditions present within the plots. Furthermore, replicates were obtained for all the soil samples collected, with each plot having four replicates. Four healthy soil samples, designated L1, L2, L3, and L4, were collected and combined to form a replicate representing the soil from the rhizosphere in the chemical fertilizer‐treated plot (G1). Similarly, samples L9, L10, L11, and L12 were used for the biofertilizer plot (G2), and samples LB1, LB2, LB3, and LB4 were used for the uncultivated bulk soil (G3), resulting in a total of 12 samples.

Following collection, the soil samples were separated, sieved, and maintained in airtight containers at 4°C. The portions of soil procured were transferred to the laboratory via an ice box and preserved for subsequent analysis at −80°C.

### 2.3 Assessment of Soil Chemical and Physical Characteristics

#### 2.3.1 Analysis of the Soil Properties of the Bulk and Rhizosphere Soils of *A. ampeloprasum*

The soil characteristics of the individual portions of the soil were investigated individually within 2 weeks of collection. For the physicochemical analysis, 20 g soil samples were crushed, desiccated, thoroughly homogenized, and subsequently subjected to a sieving process utilizing a mesh with an aperture size of 2 mm to eliminate particulate matter and organic debris (Enagbonma et al. 2020). The soil particle size was examined via a hydrometer (Mozaffari et al. 2024). The hydrogen ion concentration of the soil (pH) was determined through the use of a pH meter with deionized water to the soil mixture at a 1:2.5 ratio (Baquy et al. 2024), and the total nitrogen was evaluated following the method outlined by M. Li et al. (2022). At a pH of 7.0, via the ammonium acetate (1 M) extraction technique, the concentrations of extractable magnesium (Mg), potassium (K), calcium (Ca), and sodium (Na) were quantitatively assessed subsequent to the extraction process (Gharaibeh et al. 2021). The estimated exchangeable ions of potassium (K^+^) were determined via a flame photometer, whereas exchangeable Ca^2+^ and Mg^2+^ were quantified via an atomic absorption spectrophotometer (Suriyagoda et al. 2024). Spectrophotometry was used to determine the amount of obtainable phosphorus (P), and organic carbon was quantified via the Walkley–Black method (Cui et al. 2022). Available NO_3_^−^ and soil available NH_4_^+^ were assessed via the technique highlighted by Garmay et al. (2024). The relative proportions of the sand, clay, and silt components were estimated via the approach described by Chenu et al. (2024).

### 2.4 Metagenomic DNA Isolation, Sequencing, and Functional Characterization Analyses

To preserve the integrity of biological replication, soil samples were obtained as independent replicates from each designated plot. Each replicate was subjected to separate processing. DNA was isolated from 20 g of each individual soil replicate via the PowerSoil Pro Kit via DNeasy (Qiagen, Germany) with reference to the kit's procedure, ensuring no pooling of samples prior to sequencing. Subsequently, metagenomic libraries were individually prepared for each replicate and subjected to advanced, high‐volume sequencing via the Illumina NovaSeq X Plus platform with a paired‐end 150‐base pair (PE 150) interface (Figure S3).

The genomic DNA was preliminarily sheared to yield fragments with an average length of approximately 350 base pairs. base pairs via a sonication device (Covaris) to facilitate the construction of the sequencing library. Following fragmentation, end‐repair processes were implemented, polymerase chain reaction (PCR) amplification was executed, and adapter molecules were ligated to finalize the library preparation procedure. A comprehensive assessment was performed to determine the quality of the resulting libraries via an automated electrophoresis system (AATI) to determine the appropriate insert size distribution, whereas quantitative PCR was employed to confirm concentrations exceeding 3 nM. Libraries that met the established quality assurance standards were pooled and subsequently subjected to PE150 sequencing.

The crude sequencing reads underwent a preliminary processing phase involving the elimination of adapter sequences and sequences of poor‐quality reads through the Fastp software package (S. Chen 2023). Bowtie2 was then implemented to eliminate impure sequences from the host DNA (Langmead and Salzberg 2012). The resulting high‐quality reads were subjected to de novo assembly via MEGAHIT with meta‐large preset parameters (D. Li et al. 2016). Scaffolds containing ambiguous nucleotide junctions (denoted as “N”) were removed to generate high‐quality scaffold contigs (scafftigs) (Lei et al. 2019).

Open reading frames (ORFs) were predicted via MetaGeneMark (Mandal et al. 2024), with sequences shorter than 100 nucleotides excluded from downstream analyses. A nonredundant gene collection was constructed by employing CD‐HIT to cluster and remove redundant ORFs on the basis of sequence similarity (Commichaux et al. 2021). The quality‐filtered sequencing data were then mapped back to this gene database via Bowtie2, and genes exhibiting low read counts (≤ 2) were discarded for further analysis (Zeller et al. 2014). The prevalence of genes was normalized on the basis of both gene length and read count (Villar et al. 2015).

Taxonomic and functional characterization was achieved via alignment of unigene sequences to the Micro_NR (comprising unigene reads from archaea, viruses, and bacteria) as well as the NCBI_NR databases (https://www.ncbi.nlm.nih.gov/) (for fungal retrieval) via DIAMOND (https://github.com/bbuchfink/diamond/) (Buchfink et al. 2015), employing the BLASTP algorithm and a stringent e‐value threshold of 1e − 5. The enriched genes were further enriched via the Kyoto Encyclopedia of Genes and Genomes (KEGG) (http://www.kegg.jp/kegg/) database (Kanehisa et al. 2017; Kanehisa 2006) and the eggNOG database (http://eggnogdb.embl.de/#/app/home) (Huerta‐Cepas et al. 2016). From each alignment sequence, the best BLAST hits were chosen for subsequent in‐depth analysis (Bäckhed et al. 2015; J. Li et al. 2014; J. Qin et al. 2012; N. Qin et al. 2014). On the basis of these alignments, the prevalence of different recognized functional groups was determined. Specifically, the representation of each functional classification was quantified by aggregating the prevalence values of all genes assigned to that particular category (Karlsson et al. 2012; J. Li et al. 2014).

Species‐level taxonomic assignment was performed via the lowest common ancestor (LCA) algorithm as implemented in the MEGAN software package (https://en.wikipedia.org/wiki/Lowest_common_ancestor) (Feng et al. 2015; Huson et al. 2007; J. Li et al. 2014).

On the basis of the LCA annotation data and the corresponding gene abundance profiles, taxonomic abundance tables were generated at multiple phylogenetic levels, ranging from kingdom to species. The relative abundance of each species within a given sample was quantified by aggregating the prevalence values of all genes assigned to that species (Karlsson et al. 2012). Statistical analyses, including relative abundance summaries and abundance clustering heatmaps, were conducted via the R ade4 package (Rao 1964). Intergroup species variation was assessed via MetaGenomeSeq and LEfSe analyses, incorporating permutation testing and the LEfSe software package with an LDA score threshold of 4 (Segata et al. 2011). Diversity indices were computed via the vegan R package (Oksanen et al. 2016), and analysis of similarities (ANOSIMs) tests were performed to estimate variance between experimental groups. Data visualization was achieved via both the “R” package and Microsoft Excel (Zádrapová et al. 2024).

Finally, species selection on the basis of gradient significance at the species level was implemented via random forest analysis, which was supported by the randomForest package and R pROC (Breiman 2001). Important species were identified on the basis of metrics such as the mean decrease in the Gini coefficient and the mean decrease in accuracy. A 10‐fold cross‐validation procedure was conducted across the models, and receiver operating characteristic (ROC) curves were constructed to assess the classification performance. The sequence data pertinent to this study have been archived in the NCBI Sequence Read Archive (SRA) and can be accessed via the designated accession numbers SRP537120, SRP537121, and SRP537188.

### 2.5 Statistical Evaluation

To evaluate the variations in physicochemical characteristics, Tukey's pairwise evaluation test was used for statistical comparison via one‐way ANOVA (*p* < 0.05). The Shannon index and Pielou evenness index for the diversity metrics in each sample were evaluated, and the indices across various plots were correlated via the Kruskal–Wallis test. To evaluate the variations in community composition among the sample groups, 999 permutations were used to perform one‐way ANOSIM. This was subsequently complemented by a principal coordinate analysis (PCoA) founded on the Euclidean distance matrix to effectively depict β diversity (Jiang et al. 2022). The patterns of functional category distributions among the samples of soil from the chemical and biofertilizer plots, as well as those from the bulk soils, were visualized via a Euclidean distance matrix with principal component analysis (PCA). To evaluate which environmental parameters are fit to illustrate the composition of the functional categories, we employed redundancy analysis (RDA), Spearman's correlation coefficient, forward selection of environmental parameters, and a statistical significance analysis utilizing a Monte Carlo permutation test, which incorporated 999 random permutations. The RDA included every environmental variable mentioned in Table 1 of our previously published article (Shittu et al. 2025). The abundance clustering heatmaps were generated with the R ade4 package (Rao 1964). Similarly, with RStudio (v. 2025.09.2 + 418), Spearman's correlation matrix, PCoA, RDA, and PCA were visualized.

**Table 1:** α Diversity table illustrating the diversity assessment of microbial functional categories at EggNOG level 1, which was based on the Shannon and Pielou evenness indices across the sample plots.

Group	G1	G2	G3	*p* value
Shannon	2.793 ± 0.001	2.789 ± 0.002	2.792 ± 0.0008	0.04
Pielou's evenness	0.890 ± 0.0003	0.889 ± 0.0005	0.891 ± 0.0002	0.03

## 3 Results

### 3.1 Analysis of the Physicochemical Parameters of *A. ampeloprasum* Rhizosphere Soils Under Various Fertilization Practices and Uncultivated Bulk Soil

The summary analysis of the soil physicochemical characteristics, as stated in Table 1 of our previously published article (Shittu et al. 2025), revealed that while the other physicochemical parameters did not significantly differ across the soil samples, the amount of nitrate nitrogen (N─NO_3_) was significantly greater in the biofertilizer plots and bulk soil samples (*p* < 0.05) than in the chemical fertilizer plots (Table S1).

### 3.2 Analysis of the Functional Categories of *A. ampeloprasum* Rhizosphere Microbiomes Under Different Fertilization Practices and in Bulk Soil

The metagenomic sequence information (Table S2) previously reported in our article (Shittu et al. 2025) was used to analyze the functional categories.

**Figure 1:**
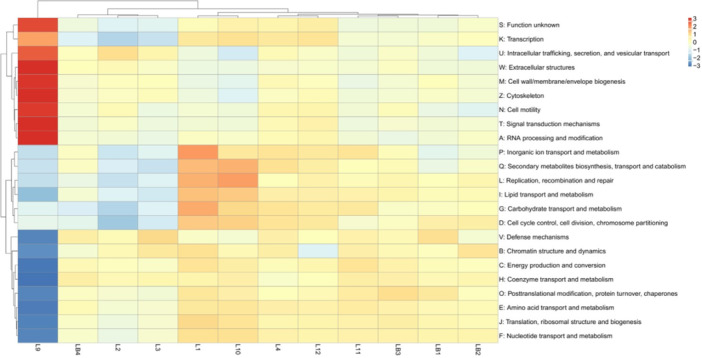
Clustered heatmap illustrating the primary functions at EggNOG level 1. The scale indicator shows a color intensity gradient that reflects the abundance level of the functional groups. G1, *Allium ampeloprasum* rhizosphere soil samples from the chemical fertilizer plot; G2, soil samples from the rhizosphere of the biofertilizer plot; G3, uncultivated bulk soil samples.

The results of EggNOG level 1 identified 23 primary functional categories associated with the bulk soil and *A. ampeloprasum* rhizosphere microbiomes. The findings indicated that 13 functional diversities were prevalent in the rhizosphere soil samples from the biofertilizer plot (G2), four functional categories were predominant in the soil samples from the rhizosphere of the chemical fertilizer plot (G1), and eight functional categories were prevalent in the uncultivated bulk soils (G3) (Figures 1 and S2 and Table S4). As revealed by our metagenome analysis, energy production and conversion (7.02%); intracellular trafficking, secretion, and vesicular transport (1.95%); and defense mechanisms (1.83%) were prevalent in the chemical fertilizer plot (G1), whereas carbohydrate transport and metabolism (5.73%); replication, recombination and repair (5.21%); transcription (5.70%); cell wall/membrane/envelope biogenesis (4.87%); inorganic ion transport and metabolism (4.96%); signal transduction mechanisms (4.81%); and secondary metabolite biosynthesis (2.89%) dominated the rhizosphere soil sample from the biofertilizer plot (G2). However, uncultivated bulk soil (G3) was dominated by amino acid transport and metabolism (8.61%); energy production and conversion (7.02%); lipid transport and metabolism (3.92%); translation, ribosomal structure and biogenesis (4.68%); coenzyme transport and metabolism (2.75%); and posttranslational modification, protein turnover, and chaperones (3.37%) (Figures 1 and S2 and Table S4).

**Figure 2:**
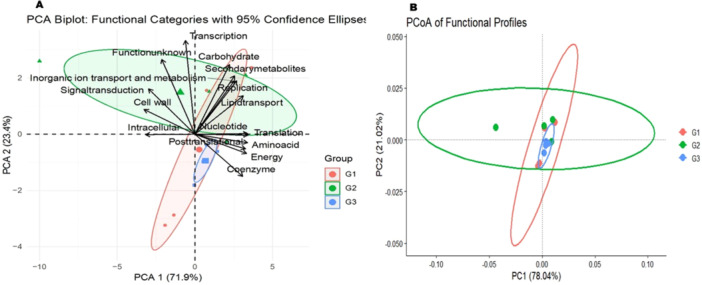
(A) Principal component analysis (PCA) chart of the top 16 microbial functional categories (relative abundance ≥ 2%) in the rhizosphere of *Allium ampeloprasum* (G1 and G2) and uncultivated Bulk soil (G3). For PCA‐Axis 1 (99.06%) and PCA‐Axis 2 (0.94%), the differences were calculated via Euclidean dissimilarities. (B) Functional category analysis of samples in rhizosphere soil (G1 and G2) and bulk soils (G3) via principal coordinate analysis (PCoA).

The analysis of functional diversity revealed that, relative to the other groups (G1 and G3), G2 presented elevated levels across multiple functional categories (*p* > 0.05) (Figure 1 and Table S7). The distributions of the functional sets within the rhizosphere and the bulk soils are depicted in the PCA biplot, which explained 95.3% of the total variation across the two axes (Figure 2A). The first two principal components (PCs) of the PCA biplot analysis (PC1 and PC2) accounted for 71.9% and 23.4% of the total variability, respectively. However, the magnitude of the PCA arrow reflects the strength of the associated functional diversity that prevails in each sample plot. The resulting PCA biplot, with 95% confidence ellipses, demonstrated distinct clustering and functional differentiation among the groups. Group G2 presented the highest confidence ellipse and the greatest distribution across both PC axes. The variation observed in group G2 was significantly correlated with essential functional diversity, including inorganic ion transport and metabolism; secondary metabolite biosynthesis, transport, and catabolism; carbohydrate transport and metabolism; cell wall/membrane/envelope biogenesis; signal transduction mechanisms; unknown functions; transcription; and replication, recombination, and repair.

In contrast, Group G3 presented the most restricted clustering or the least functionally specialized group, suggesting a lower diversity of functional categories. Group G1 presented moderate variation and was associated primarily with functions related to energy production and conversion; translation, ribosomal structure, and biogenesis; coenzyme transport and metabolism; and amino acid transport and metabolism.

**Figure 3:**
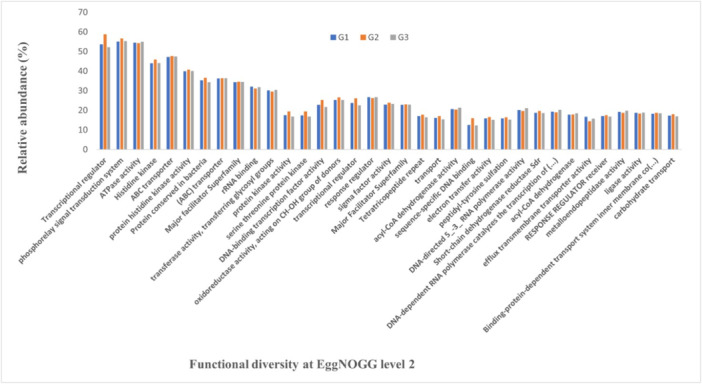
Abundance levels of functional diversity at EggNOGG level 2.

At EggNOG level 2, the chemical fertilization plot rhizosphere samples (G1) were dominated by rRNA binding (32.07%), followed by acyl‐CoA dehydrogenase activity (20.64%) and DNA‐directed 5_–3_RNA polymerase activity (20.17%), whereas the transcriptional regulator (58.79%), phosphorelay signal transduction system (56.63%), histidine kinase (45.95%), ATP‐binding cassette (ABC) transporter (47.71%), protein histidine kinase activity (40.78%), protein conserved in bacteria (36.64%), and major facilitator superfamily (MFS) (34.66%) dominated G2 (biofertilizer plot). Bulk soil samples were found to be dominated by ATPase activity (54.99%), followed by ABC transporters (36.38%), transferase activity, glycosyl group transfer (30.42%), and response regulators (26.77%) (Figure 3 and Table S5).

**Figure 4:**
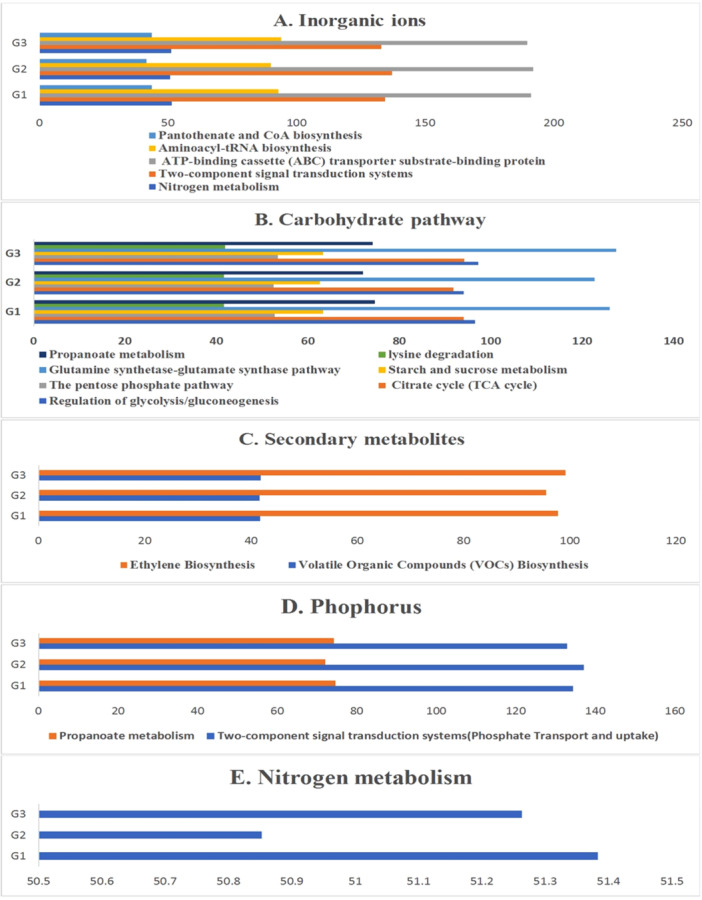
Abundance levels of the (A) inorganic ion, (B) carbohydrate, (C) secondary metabolite, (D) phosphorus, and (E) nitrogen pathways obtained from KEGG Orthology in soil samples from chemical fertilizer and biofertilizer plots and bulk soil. CoA, coordinate analysis; KEGG, Kyoto Encyclopedia of Genes and Genomes; TCA, tricarboxylic acid; tRNA, transfer RNA.

The level 3 KEGG pathway database was used to analyze our metagenome for nutrients and metabolic pathways in the soil samples. Arginine biosynthesis; valine, leucine, and isoleucine degradation; lysine degradation; and arginine and proline metabolism from the amino acid metabolic pathway were all dominant in the G3 plot, whereas glycine, serine, and threonine metabolism and tryptophan metabolism were dominant in the G1 plot. In the carbohydrate pathway, arginine and proline metabolism; regulation of glycolysis/gluconeogenesis; the citrate cycle (tricarboxylic acid [TCA] cycle); the pentose phosphate pathway; the glutamine synthetase‐glutamate synthase pathway; and lysine degradation were all dominant in the G3 plot, whereas starch and sucrose metabolism and amino sugar and nucleotide sugar metabolism were dominant in the chemical fertilizer plot. Propanoate metabolism was also dominant in the G1 plot. However, two‐component signal transduction systems, ABC transporter substrate‐binding proteins from the inorganic ion pathway, and two‐component signal transduction systems (phosphate transport and uptake) from the phosphorus pathway were dominant in the G2 plot two‐component signal transduction systems (phosphate transport and uptake) (Figure 4 and Table S6).

### 3.3 Estimates of the β and α Diversity of the Functional Diversities of Bulk and *A. ampeloprasum* Rhizosphere Soil Samples

The α diversity across functional diversities at EggNOG level 1 was assessed via the evenness and the Shannon indices (Table 1). The Shannon and evenness indices among the samples were significantly different when analyzed with the Kruskal–Wallis test at *p* < 0.05, and the evenness indices and Shannon diversity indices fell within the expected normative range (Table 1). Their values, ranging from 2.789–2.793 and 0.889–0.891, respectively, suggest a moderate level of functional diversity. These findings indicate that functions are both prevalent and moderately distributed within microbial communities across rhizosphere samples in relation to those in bulk soil.

The beta diversity of the soil samples was visualized via PCoA according to their relative abundance (Figure 2B). The first principal component (PC1) captures approximately 78.04% of the total variance present in the data set, whereas the second principal component (PC2) accounts for an additional 21.02% of the variance. Collectively, these two components capture 99.06% of the overall variance, thereby providing an almost exhaustive representation of the underlying multivariate structure of the data set (Figure 2B). The various functional categories found in the soil samples did not significantly differ, as indicated by ANOSIM, which revealed a *p* value of 0.09 and an *R* value of 0.15. The position occupied by G1 on the PCoA plot is clearly distinct from that of both G2 and G3, indicating clear differences in functional categories among these groups. Moreover, G2 and G3 are separated from each other, displaying distinct functional variations. However, the samples within each group (G1, G2, and G3) clustered closely together, suggesting similarities and consistent functional characteristics within the groups. However, G2 exhibited distinctive functional categories that were notably different from those of G1 and G3, highlighting the unique functional activities specific to this group (Figure 2B).

### 3.4 Impact of Soil Attributes on the Diversity of Microbial Functions in Soil Samples

**Figure 5:**
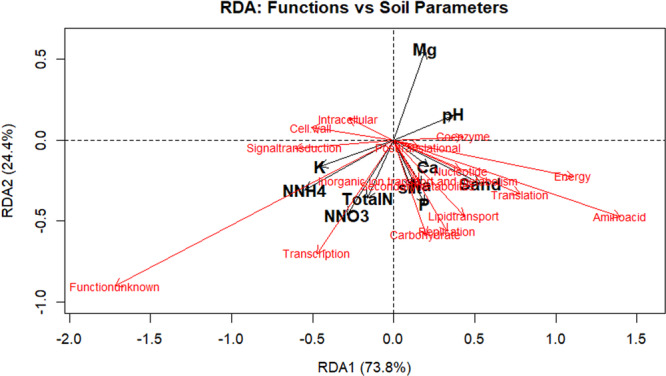
Redundancy analysis (RDA) ordination displaying the associations between the soil physicochemical attributes and the distributions of the top 16 functional categories (relative abundance ≥ 2%) in the soil samples. The vectors delineate both the orientation and magnitude of the influence of the soil variables.

**Figure 6:**
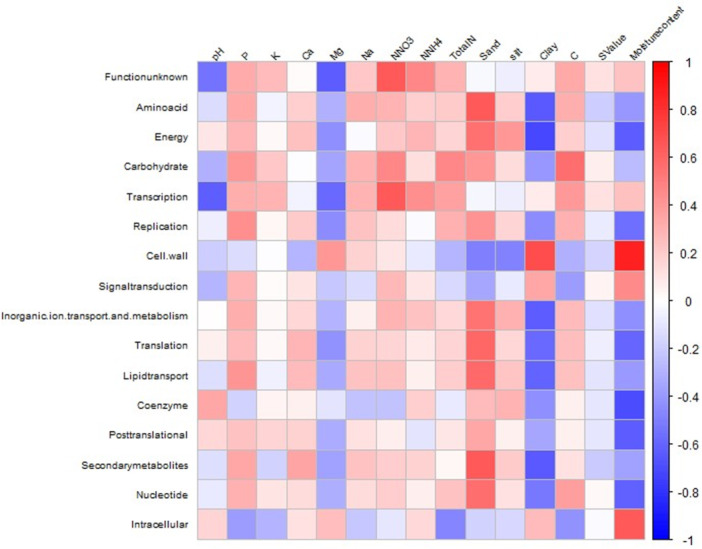
Spearman correlation analysis of microbial functional categories and soil physicochemical parameters.

Spearman correlation analysis (Figure 6 and Table S8) was used to assess the pairwise monotonic relationships between key soil physicochemical parameters, as summarized in Table 1 of our previously published article (Shittu et al. 2025), and microbial functional categories, thereby identifying significant positive and negative associations, as indicated by correlation coefficients and significance values. Subsequently, RDA (which accounted for 73.8% on axis 1 and 24.4% on axis 2) was employed to evaluate the comprehensive impact of these physicochemical parameters on the microbial functional categories (Figure 5).

Several soil physicochemical factors, including potassium (K), ammonium nitrogen (N–NH_4_), nitrate nitrogen (N─NO_3_), calcium (Ca), sodium (Na), and total nitrogen, are associated with microbial functional assemblages, indicating their potential influence on functional diversity (*p* > 0.05). The soil physicochemical parameters identified from forward selection, which effectively explained the variations, indicated that carbon was more influential than the other properties (Figure S4). Carbon presented a *p* value of 0.17, coupled with a 99% contribution to the variation, which corresponds to the percentage explained (Table 2). The RDA results revealed that signal transduction was positively correlated with potassium (K) and that transcription was positively correlated with total nitrogen and nitrate nitrogen (N─NO_3_) (rho = 0.16, *p* < 0.05). Similarly, energy production and conversion; amino acid transport and metabolism; lipid transport and metabolism; carbohydrate transport and metabolism; nucleotide transport and metabolism; and inorganic ion transport and metabolism were positively correlated with calcium (Ca), phosphorus (P), sodium (Na), and silt (Figures 5 and 6). However, intracellular trafficking, secretion, vesicular transport, and cell wall/membrane/envelope biogenesis were negatively correlated with sodium (Na), N─NO_3_, and total nitrogen (total N) contents.

**Table 2:** Forward selection result of environmental variables explaining microbial functional composition in redundancy analysis (RDA).

Name	Explains (%)	Pseudo‐*F*	*p*
C	99.02	96.8	0.17
Moisture content	1.0	< 0.1	1.

### 3.5 Comparative Taxonomic Profiling of the Rhizosphere Microbiome of *A. ampeloprasum* and Bulk Soil

**Figure 7:**
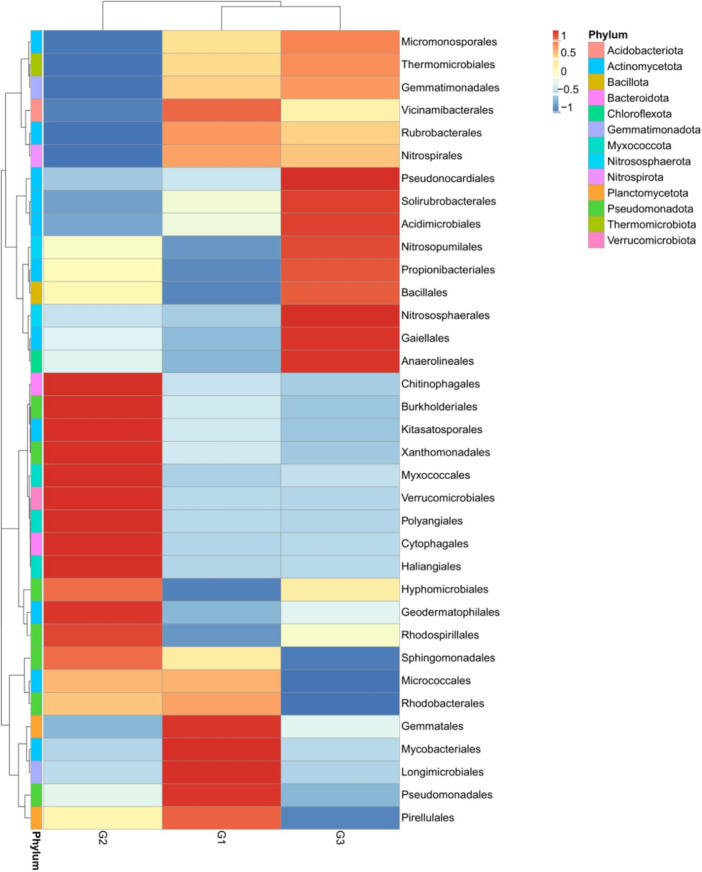
Heatmap illustrating the major taxa at the order level. The scale indicator shows a color intensity gradient that reflects the abundance level of the functional groups. G1, *Allium ampeloprasum* rhizosphere soil samples from the chemical fertilizer plot; G2, soil samples from the rhizosphere of the biofertilizer plot; G3, uncultivated bulk soil samples.

The microbial composition of the examined soil samples was dominated by bacteria, accounting for approximately 98% of the total observed microorganisms. At the order level, the chemical fertilizer plot (G1) was dominated by Micrococcales (6.49%), Rubrobacterales (2.06%), and Nitrospirales (1.15%), whereas the biofertilizer plot (G2) was dominated by Cytophagales (1.92%), Hyphomicrobiales (5.52%), Polyangiales (1.62%), Verrucomicrobiales (1.62%), and Kitasatosporales (2.30%). The bulk soil plot was dominated by Solirubrobacterales (4.65%), Gaiellales (2.05%), and Nitrososphaerales (1.47%). Among the microbial communities analyzed, only Vicinamibacterales and Gemmatimonadales were significantly different (*p* < 0.05), whereas the remaining microbial taxa were not significantly different (*p* > 0.05) (Figure 7 and Table S3).

## 4 Discussion

In this work, we examined and profiled the functional profiles of the microbial community in the rhizosphere of *A. ampeloprasum* under different fertilization systems compared with those in the bulk soil via a shotgun metagenomic approach. Compared with other techniques, such as amplicon sequencing, shotgun metagenomic sequencing has several benefits for examining the functional diversity of microbial communities. It offers a more reliable resolution and detailed comprehension of microbial diversity and functional characteristics (Bars‐Cortina et al. 2024; Fadiji et al. 2021b). In the samples, the microbial orders found to be most dominant were Propionibacteriales (G3), Micrococcales (G1), Hyphomicrobiales (G2), Cytophagales (G2), Solirubrobacterales (G3), Burkholderiales (G2), and Sphingomonadales (G2) (Figure 7 and Table S3). These microorganisms play a vital role in the cycling of essential elements, increasing plant development, and facilitating other helpful relationships between plants and microbes (Ling et al. 2022). These findings suggest that the rhizosphere of *A. ampeloprasum* is a thriving habitat that encourages its proliferation and function.

EggNOG functional analysis at levels 1 and 2 was employed to infer the functions of the identified rhizosphere microbiomes under different fertilizer applications and bulk soil samples (Figures 1 and 3 and Tables S4 and S5), whereas KEGG orthology at level 3 (Figure 4) was employed to examine the metabolic pathway. In this context, the EggNOG database offers a range of orthologous categories at different taxonomic levels, along with functional annotations and cross‐domain orthology information of environmental samples (Hernández‐Plaza et al. 2023). EggNOG level 1 analysis revealed several dominant functional categories associated with metabolism, including both catabolic and anabolic processes. However, the EggNOG level 1 analysis revealed a moderate distribution of functional composition across the fertilized plot and bulk soil (*p* > 0.05). The analysis revealed that the samples of soil from the *A. ampeloprasum* rhizosphere from the biofertilizer plots (G2) presented the highest microbial functional diversity, followed by the uncultivated bulk soils. In contrast, the rhizosphere soils from the chemical fertilizer plot (G1) presented the lowest microbial functional diversity (Figures 1 and S2 and Table S4), as reported in this study. This finding supports the idea that bacteria are grouped according to functional rather than taxonomic affiliation (Khan et al. 2024). This work also indicates that analyses of functional genes, rather than “species,” may be the primary scale at which to approach the organization and composition of microbial communities. The functional roles of rhizosphere microbiomes are also dependent on host type and environmental factors rather than their taxonomic classification, according to Xun et al. (2024). The rhizosphere soils from the biofertilizer plot (G2) presented more prevalent functional categories, as indicated by this study (Figures 1 and S2 and Table S4). These findings are consistent with those of Lin et al. (2021), who reported that biofertilizer treatment enriched the rhizosphere microbiome and functional diversity, such as lipid metabolism, protein transport, replication and repair, and carbohydrate metabolism, in the sugarcane rhizosphere. Similarly, Enebe and Babalola (2022) demonstrated that maize rhizosphere soil cultivated under organic fertilizers enhances functional categories associated with sulfur, protein, phosphorus, and secondary metabolism. Moreover, Fadiji et al. (2021a), through shotgun metagenomic analysis of maize root‐associated endophytic microbiomes from various farm sites under organic and inorganic fertilization, revealed functional diversities related to the metabolism of carbohydrates, nitrogen, aromatic compounds, DNA, and fatty acids. These findings showed that the various communities of microorganisms found in the root maize plants are highly active, contributing to the improved health of the plants.

Some of the microbial orders identified in this study have been previously reported to harbor a broad spectrum of metabolic functions in plant systems, particularly those associated with carbohydrate, nitrogen, and phosphorus metabolic pathways. These include Burkholderiales in rice (Suresh and Rameshkumar 2026), Pseudomonadales in *Angelica sinensis*, Bacillales in *Lonicera macranthoides* (Zhang et al. 2026), and Sphingomonadales/Sphingopyxis in *Brassica napus* (N. Li et al. 2026). Similarly, O. Omotayo et al. (2021) reported that certain groups of microorganisms, specifically those in the orders Pseudomonadales, Myxococcales, Xanthomonadales, and Burkholderiales, are strongly linked to important metabolic functions in the rhizosphere of maize. These activities include the processing of fats and related compounds, energy production, the creation of amino acids and related substances, the capture of light energy, and the cycling of nitrogen.

These bacterial orders perform vital functions in the soil environment by facilitating nutrient turnover and decomposing intricate organic substances. This activity promotes plant vigor and contributes to the overall health and stability of the soil ecosystem. Furthermore, these groups exhibit synergistic metabolic interactions that support essential processes, such as increasing phosphorus accessibility, the production of growth hormones from tryptophan, and the decomposition of environmental contaminants, such as polychlorinated biphenyls and dioxins. Our metagenomic study additionally revealed the existence of several notable archaeal and fungal microbial orders. Specifically, we identified members of Nitrososphaerales and Nitrosopumilales (within the phylum Thaumarchaeota), as well as Sordariales (within the phylum Ascomycota).

Baz (2026) identified members of the phylum Thaumarchaeota, encompassing representatives of the orders Nitrososphaerales and Nitrosopumilales, as the predominant archaeal taxa inhabiting the rhizosphere of *Moringa oleifera*. It was found to participate in key biogeochemical processes, notably nitrogen and sulfur metabolism, carbon transformation pathways, and cellular responses to oxidative stress. Similarly, members of the fungal order Sordariales, including families, such as Chaetomiaceae, have been reported to exhibit extensive secondary metabolism, producing diverse classes of metabolites such as alkaloids, polyketides, peptides, and terpenes that display pronounced bioactivities with potential applications in plant protection and growth regulation (Ibrahim et al. 2021).

These rhizosphere microbiomes have also been linked to different key functions, including nitrogen cycling, the breakdown of organic materials, and the enhancement of nutrient availability (Daraz et al. 2024; Devkota et al. 2024; O. Omotayo et al. 2021). These functions play crucial roles in fostering plant development and sustaining overall soil quality.

Our alpha diversity analysis (Shannon and evenness indices) of the samples was confirmed with a Kruskal–Wallis test (*p* < 0.05) (Table 1). The Shannon diversity index value indicates that the functions expressed by the microbes at each plot nearly approached the theoretical limit of 2.81, suggesting that nearly all functions are found in the portions of the soil from all the sites (Fadiji et al. 2021a). The evenness values of the functions for the samples of soil were low, indicating that certain microbial orders were enriched in relation to microbial functions and rhizosphere stability. The Shannon index values indicated that G2 had some variation from the other groups (G1 and G3). In the biofertilizer plot, both the Shannon diversity index and Pielou's evenness index were lower, indicating that the community was strongly dominated by a few taxa. However, these dominant taxa included both specific taxonomic and functional groups favored by the biofertilizer, suggesting that the functional dominance of key groups can sustain ecosystem functioning even when overall diversity and evenness are reduced. This finding is consistent with that of M. Wang et al. (2022), who reported that community productivity and multifunctionality are often more strongly driven by functional dominance and the performance of particular functional groups than by taxonomic diversity alone.

As revealed by the PCoA plot based on Bray–Curtis dissimilarity, the functional categories formed a partial separation among groups, with the first two axes (1 and 2) of the PCoA graph explaining 99.06% of the total variation (Figure 2B). The PCoA revealed partial clustering among the three groups along PC1 (78.04% variance), indicating that fertilization treatment modulated rhizosphere microbial functional diversity, with biofertilizer application inducing additional, moderate, and consistent shifts in functional categories which correlated with improved plant growth (Figure 2B).

However, one replicate (L9) within the biofertilizer‐treated plot (G2) is distinctly separated from the clustered samples, which may be influenced by the enrichment of specific functional categories. The differences observed in the biofertilizer plot (G2) along PC1 suggest that biofertilizer influences the functions of fine root stimulation, enhanced metabolite release, and nutrient flow, and thereby supports enriched functional microbial categories (Mącik et al. 2023). This aligns with the findings of Cheng et al. (2025), who reported that biofertilizers enhanced soil microbial activity and metabolic functions, such as nutrient cycling and carbon metabolism, more effectively than chemical fertilizers.

Although the separation did not alter the significant overall group effect (ANOSIM: *p* = 0.09 and *R* = 0.15), it underscores the importance of biological replication in capturing such variability, which could represent extreme positive responses informative for field‐scale optimization. The nonsignificant responses in the functional categories might indicate that the soil chemistry and plant developmental stage exert stronger influences on microbial functional categories than microbial inoculation alone does, emphasizing the need to consider these factors when evaluating biofertilizer impacts (M. Li et al. 2023; J. Wang et al. 2024).

Our analysis via a PCA biplot (Figure 2A) reveals a pattern that accounts for 95.3% of the total variance across the two principal axes. The PCA plot suggests that the application of biofertilizer may have fostered broader functional capabilities in G2 than in the other investigated groups, G1 and G3. G2 not only demonstrated alignment with unique functional categories, such as inorganic ion transport and metabolism; signal transduction mechanisms; cell wall/membrane/envelope biogenesis; and transcription; and unknown functions but also overlapped with G1 in core metabolic functions, such as carbohydrate transport and metabolism; biosynthesis, transport and catabolism of secondary metabolites; replication, recombination, and repair; lipid transport and metabolism; and translation, ribosomal structure, and biogenesis. These findings suggest that biofertilizer applied in G2 influenced microbial communities and their functional diversity. This ultimately represents a more extensive functional profile. However, group G1 has a more pronounced focus on the cycling of nutrients, amino acid transport and metabolism, coenzyme transport and metabolism, and energy‐related activities, whereas the functional properties of the G3 group remain less defined. Consequently, the observed functional distribution supports our hypothesis that fertilizer input may have increased microorganism adaptability within the G2 group, bolstering both metabolic and adaptive mechanisms within the rhizosphere.

This result is consistent with previous research by Enebe and Babalola (2022), who documented comparable functional enrichments in the maize rhizosphere in a soil under the influence of organic and inorganic fertilizer treatments. The variance observed among the soil samples indicates that the microbial metagenomes in the rhizosphere are functionally specialized, reflecting distinct metabolic roles. Within the PCA chart (Figure 2A), each point corresponding to a single metagenome represents the proportional composition of sequences associated with specific functional categories. Concurrently, the vector arrows illustrate the impact and relative contribution of individual functional categories to the spatial arrangement of the samples. On the basis of these findings, making predictions becomes easier as to which metabolisms are important to the rhizosphere microbiome on the basis of the functional categories identified in each sample plot.

For example, the biofertilizer plot was enriched in terms of the abundance of secondary metabolite biosynthesis, transport, and metabolism; carbohydrate transport and metabolism; and inorganic ion transport and metabolism, which were abundant and specific to the biofertilizer plot (G2).

Our results align with those of Jain et al. (2026), who demonstrated that Jeevamrit biofertilizer formulations exhibit a COG distribution with increased gene allocation to the metabolism of carbohydrates, nitrogen, iron, sulfur, aromatic compounds, and potassium, which are associated with diverse plant‐beneficial microbes. Similarly, Falcón‐Piñeiro et al. (2026) reported the predominance of genes related to the transport and metabolism of inorganic ions, lipids, coenzymes, nucleotides, carbohydrates, and amino acids in a novel biofertilizer strain (*Bacillus altitudinis* GG‐22) when applied to olive trees. Since biofertilizer plots (G2) are known to contain relatively high levels of organic matter and beneficial microorganisms, plants growing there are predicted to have relatively high levels of carbon (Fadiji et al. 2021a). Rhizosphere microbiomes grow in this area because numerous exudates secreted by plant roots in the rhizosphere serve as microbe attractants. These root exudates are essential in determining how microbes and plants interact in the rhizosphere. These various substances, which include natural metabolites, acids of organic and amino group origin, and carbon‐based sugars, facilitate communication between microbes and plants (Bharadwaj et al. 2025). Consequently, increased metabolism of fats and carbohydrates, with the biosynthesis, transport, and breakdown of secondary metabolites from the biofertilizer plot (G2), as revealed from these findings align with those of Fadiji et al. (2021a) and Enebe and Babalola (2022), where the production and transport of secondary metabolites, such as fatty acids, lipids, and isoprenoids in plants, as well as the abundance of potassium and carbohydrate metabolism and secondary metabolism, are stimulated by organic fertilizers.

Carbohydrates are vital for plant energy and growth. Microorganisms support carbohydrate metabolism by ensuring that plants have the necessary nutrients for photosynthesis and energy production. In turn, efficient carbohydrate transport and metabolism help in the distribution of energy throughout the plant, which helps support development (Su et al. 2024). The abundance of sequences associated with important metabolic pathways related to carbohydrate transport and metabolism, such as the citrate cycle (TCA cycle), the regulation of glycolysis/gluconeogenesis, the metabolism of starch and sucrose, propanoate, and the metabolism of amino sugars and nucleotides, further supported these findings at lower levels (KEGG level 3) (Figure 4 and Table S6).

The relatively high inorganic ion transport and metabolism observed in the biofertilizer plot (G2) are in agreement with the findings of Xu et al. (2021), where high inorganic ion transport and metabolism were reported in drought‐induced rhizosphere soils. Inorganic ion transport and metabolism sequester ions, such as nitrogen, potassium, and phosphorus, which are crucial nutrients for the growth of plants, and the solubilization and mobilization of these essential nutrients increase the accessibility of these ions to plants, facilitating their uptake and metabolism (Bhatla and Lal 2023). These genes are expressed at relatively low levels (KEGG level 3) via several metabolic pathways, such as nitrogen metabolism, two‐component signal transduction systems, ABC transporter substrate‐binding protein, aminoacyl‐tRNA biosynthesis, and pantothenate and CoA biosynthesis.

The abundance of sequences associated with secondary metabolites was also recorded in the biofertilizer plot (G2). These findings are consistent with those of Cho et al. (2026), who reported that inoculating soybean roots with rhizospheric bacteria significantly increased the production of secondary metabolites, specifically isoflavones, phenolics, and flavonoids. Similarly, J. Ma et al. (2026) demonstrated that the biofertilizer strain *B. licheniformis* LCDD6 produced significant amounts of secondary metabolites, such as bacillibactin and indole‐3‐acetic acid, which promoted plant growth and enhanced nutrient acquisition. The metabolism of secondary metabolites such as flavonoids, alkaloids, and terpenoids helps plants defend against diseases and pests. Microorganisms can influence the production of these compounds by providing necessary nutrients and promoting plant robustness and performance (Bano et al. 2023; Lv et al. 2024). This was later rooted at lower levels (KEGG level 3) by the presence of several metabolic processes, such as volatile organic compound biosynthesis and ethylene biosynthesis.

Sequences associated with cell motility were also observed in the biofertilizer plot (G2) samples. These results align with those of X. Wu et al. (2023), where the genes encoding bacterial flagella assembly, cell motility proteins, and chemotaxis genes are highly enriched in rhizosphere soil, according to a metagenome comparative analysis of cucumber and wheat field sites. By improving networking, movement, and control of nutrient acquisition inside the host, motility can help rhizosphere bacteria perform better (X. Wu et al. 2023) and improve biofilm formation (P. Qin et al. 2023).

Sequences such as those related to nucleotide transport and metabolism, amino acid transport and metabolism, and coenzyme transport and metabolism were more dominant in the bulk soils (G3) than in the biofertilizer plots (G2) and chemical fertilizer plots (G1). However, these values did not differ significantly across the sites (*p* > 0.05). Our findings align with those of S. Wu et al. (2022), who identified bulk soil as a key hotspot for nitrogen cycling‐related microbial functions in biofertilized oilseed rape systems. As a result, bulk soil played a more prominent role in driving nitrogen transformation processes, thereby improving nutrient cycling efficiency.

Bulk soils are generally known as more nutrient‐limited soils than rhizosphere soils (Mueller et al. 2024). Innate microorganisms in bulk soils often rely on their own metabolic capacity to synthesize nucleotides and amino acids due to the scarcity of easily accessible organic substrates (Kaur et al. 2024; Ling et al. 2022), and the scarcity of these accessible organic substrates drives the need for enhanced transport and metabolic pathways to efficiently utilize any available resources (Kaur et al. 2024). These genes were later confirmed at lower levels (KEGG level 3) by the presence of several metabolic pathways, such as arginine biosynthesis; glycine, serine, and threonine metabolism; valine, leucine, and isoleucine degradation; lysine degradation; tryptophan metabolism; and arginine and proline metabolism (Figure 4 and Table S6).

Chemical fertilizer plots (G1) and bulk soil plots (G3) can be linked with amino acid transport and metabolism, coenzyme transport and metabolism, and nucleotide transport and metabolism.

Our results align with those of Y. Ma et al. (2022), who reported that nitrogen fertilization in rice significantly upregulated several metabolic pathways and increased the accumulation of nitrogen‐related metabolites, such as amino acids and secondary compounds.

The synthesis of nucleotides, amino acids, proteins, and histones, the fundamental building blocks of chromatin, requires nitrogen as a major nutrient. Chemical fertilizer must provide nitrogen, which is a necessary nutrient for the production of chromatin (K. Wu et al. 2020). An adequate supply of nitrogen can increase the production of nucleic acids and proteins, which aid in the structure and assembly of chromatin during cell division and growth (Ali et al. 2025; Robert and Jeronimo 2023). These effects were later elucidated at lower levels (KEGG level 3) via identifiable metabolic pathways, such as arginine biosynthesis; glycine, serine, and threonine metabolism; valine, leucine, and isoleucine degradation; lysine degradation; tryptophan metabolism; and arginine and proline metabolism (Figure 4 and Table S6).

Our results also revealed that chromatin structure and dynamics were more dominant in the chemical fertilizer plot G1 than in the other plots. Chromatin is a complex of proteins and DNA that produces chromosomes within cells (Sigismondo et al. 2022). Its structure is highly dynamic, allowing for the expression of genes in response to stressors from the environment (W. Wang and Sung 2024). This regulation is essential for microbial adaptation and survival in various soil environments (De Pedro‐Jové et al. 2023). This helps microbes optimize their metabolic activities to utilize available nutrients efficiently and adapt to changes in soil conditions, such as pH, moisture, and nutrient availability, which are crucial for maintaining microbial functions in the rhizosphere (Mohanty et al. 2023). Chromatin modifications can also influence microbial traits that affect plant–microbe interactions, such as the production of enzymes, antibiotics, and signaling molecules, which are pivotal for the health of plants as well as the fertility of the soil (Q. Chen et al. 2024).

Our metagenome analysis also revealed that the biofertilizer plot (G2) primarily contained numerous functional genes with unknown functions, which were present in the clustering‐based sequences. The high occurrence of clustered genes with unknown functions at EggNOG level 1 indicates the existence of numerous noteworthy functional genes in rhizosphere microbiomes whose roles have not yet been investigated or identified (Figures 1 and S2 and Table S4). It is imperative to acknowledge the potential importance of these genes within the rhizosphere and its microbiome. Specifically, these genes might be highly important in the areas of nutrient acquisition and mobilization, plant growth and development, and resistance to diseases and environmental challenges. The yet‐to‐be‐discovered functions of these genes hint at a wealth of new biological pathways and functions that could be exploited for advancements in agriculture, including improvements in the effectiveness of biological fertilizers toward achieving agricultural sustainability and SDGs 2 and 12.

To cope with fluctuating nutrient availability, particularly phosphate solubilization and nitrogen fixation, microorganisms employ sophisticated regulatory mechanisms as revealed by our metagenome at EggNOG level 2 (Figure 3). A central aspect of this adaptation is the phosphorelay signal transduction system, which is based on the interaction of histidine kinases and response regulators. An example is the two‐component PhoR/PhoB system, which is vital for orchestrating the cellular response to phosphate deficiency. This system allows rhizosphere microbiomes to thrive under low‐phosphate conditions by adjusting the expression of genes involved in the release of bound phosphorus (Bruna et al. 2024). In addition to solubilization, the assimilation of nutrients relies on transporters such as ABC transporters and proteins belonging to the MFS, which are critical constituents of phosphate uptake mechanisms (Shin and Cho 2024). Furthermore, gene expression is finely tuned by transcriptional regulators, including DNA‐binding transcription factors, to coordinate not only phosphate solubilization but also functions, such as nitrogen fixation (D. Das et al. 2022; Z. Wang, Zheng, et al. 2023). This intricate regulatory network ensures the efficient acquisition of phosphate from the soil. Compared with chemical fertilizers (G1) and bulk soils, biofertilizers had a greater influence on all these functions in the biofertilizer plot (G2). Pathways such as two‐component signal transduction systems (phosphate transport and uptake) and propanoate metabolism, which drive the mineralization and solubilization of insoluble phosphate, are rooted in pathways at KEGG level 3 (Figure 4 and Table S6).

Similarly, energy production and conversion; inorganic ion transport and metabolism; transcription; replication, recombination, and repair; and signal transduction mechanisms, which are highly abundant in the biofertilizer plot (G2), are associated with nitrogen fixation in our metagenomes. The high abundance observed in biofertilizer‐treated soils is due to their ability to improve the physical and chemical characteristics of the soil.

This is achieved by improving soil biological activity, enriching soil with organic matter, and facilitating access to key nutrients, including nitrogen, phosphorus, and potassium. Similarly, biofertilizer creates a more favorable environment for microbial development and activity to favor essential microbial processes, such as ion transport, gene regulation, and intercellular communication, ultimately contributing to overall soil quality and improved plant development (Ghimirey et al. 2024). Nitrogen fixation represents a vital biological transformation in which atmospheric nitrogen (N_2_), a largely unreactive molecule inaccessible to plants, is converted into ammonia (NH_3_). This process is mediated by specific microorganisms (diazotrophs) employing a sophisticated enzymatic system known as nitrogenase. The substantial energy demands of this reaction via energy production and conversion necessitate the breakdown of approximately 16 ATP molecules for each N_2_ molecule, underscoring the critical role of effective energy generation and utilization within nitrogen‐fixing bacteria (Alleman and Peters 2023). Similarly, the transport and metabolism of inorganic ions help in the process of nitrogen fixation by facilitating the absorption and transfer of nitrogen‐containing substances (Y. Li et al. 2024). Furthermore, transcription and replication, recombination and repair stringently govern the expression of nitrogenase and associated proteins in response to environmental signals, such as the prevalence of oxygen and nitrogen. Signal transduction mechanisms additionally ensure that nitrogen fixation activity is calibrated to cellular requirements and circumstances, whereas alterations after translation aid in the construction and maintenance of the functional nitrogenase system (Rivier et al. 2023; W. Wu et al. 2025). The resulting ammonia is subsequently assimilated into amino acids via biochemical pathways, promoting the development of both microbes and plants. This complex interplay of energy use, genetic control, and nutrient transport highlights the importance of nitrogen fixation as a fundamental mechanism in ecosystem nutrient dynamics and agricultural output (Y. Ma et al. 2025; Ortiz‐Medina et al. 2023). The aforementioned functions were established through pathways such as nitrogen metabolism, two‐component signal transduction systems, ABC transporter substrate‐binding protein, aminoacyl‐tRNA biosynthesis, and pantothenate and CoA biosynthesis at the lower level (KEGG level 3) (Figure 4 and Table S6).

The increased abundance of plant growth‐promoting functions related to phosphorus availability and nitrogen fixation observed in our metagenomic data is consistent with findings from previous studies, which are consistent with those of Mącik et al. (2023), who used PICRUSt‐based functional prediction and reported that applying phosphorus biofertilizer increased the abundance of functional sequences associated with metabolic activities and cellular processes, especially those involved in phosphorus pathways. Similarly, Y. Wang et al. (2021) demonstrated that bacterial inoculants increased the abundance of genes related to both inorganic phosphorus solubilization and organic phosphorus mineralization and observed a general increase in genes associated with nitrogen metabolism.

Biofertilizers introduce functionally active microorganisms with genes for nitrogen and phosphorus metabolism. Once these microorganisms establish themselves in the soil, they multiply under favorable rhizosphere conditions. This enrichment increases the abundance of nitrogen‐ and phosphorus‐related functional genes both directly, through the contribution of the inoculated strains, and indirectly, by stimulating native microbial communities (Torres et al. 2026; Xiao et al. 2026).

Spearman correlation analysis (Figure 6 and Table S8) revealed notable associations between microbial functional categories and soil physicochemical parameters. Amino acid transport and metabolism were positively correlated with Na (rho 0.32) and N─NO_3_ (rho 0.29), and transcription was also positively correlated with N─NH_4_ (rho 0.4), suggesting that microbial activity related to nutrient cycling and metabolism may be influenced by soil fertility. This is further illustrated in the RDA biplot (Figure 5), which was used to determine the effects of various soil characteristics on the functions of the microbial community. The variance between the different soil samples from the fertilized plots and the bulk soil revealed that key predicted functions of the rhizosphere microbiome are influenced or affected by soil treatments under different fertilization systems.

RDA revealed that energy production and conversion; amino acid transport and metabolism; lipid transport and metabolism; carbohydrate transport and metabolism; nucleotide transport and metabolism; and inorganic ion transport and metabolism were positively correlated with calcium (Ca), phosphorus (P), sodium (Na), and silt. However, Spearman's correlation analysis further provided insight into individual pairwise relationships. Similarly, at EggNOG level 1, the RDA through forward selection revealed that moisture content and carbon were the best predictors of the composition of the functional category, with carbon accounting for 99.02% of the total variability (Figure S4 and Table 2).

Given that physicochemical parameters are considered key factors in explaining significant portions of the differences in soil microbial structure, diversity, and functional diversity, it has been acknowledged that environmental factors serve as the main factors influencing the allocation of soil bacteria with functional diversity (Enagbonma et al. 2019; Y. Wang, Li, et al. 2023). The distribution of microbial functional categories across the fertilizer‐treated and bulk soils along the RDA axes highlights the differential functional responses influenced by the soil physicochemical parameters. These observations indicate that microbial functional diversity is largely influenced by soil physicochemical parameters, which exert considerable influence on microbial functional assemblages. This underscores the critical function of environmental circumstances in molding the configuration and variety of soil microbial communities in agricultural settings.

This demonstrated that the abundance levels of bacterial functional diversity in both environments were influenced by the soil physicochemical parameters in this investigation (Akinola et al. 2021; Enebe and Babalola 2022). Our results are in accordance with those of Nwachukwu et al. (2021), whose work showed that physicochemical parameters, through the influence of fertilizer application, affect soil microbial functional groups. Considering the functional groups highlighted in this research, soil characteristics have been demonstrated to impact the role of the microbiome functional categories in the rhizosphere of *A. ampeloprasum*.

The greater relative abundance of functions observed in the soil treated with biofertilizer (G2) than in that treated with chemical fertilizer reflects the combined effects of the rhizosphere microbiome. This highlights the dynamic interaction between rhizosphere microbiomes and plants. Different microbial metabolic pathways facilitate different functions, which in turn strengthen plant–microbe interactions and microbe–microbe communications (Babalola et al. 2025). These factors increase nutrient acquisition, stress tolerance, and overall agricultural sustainability under biofertilizer treatment. Biofertilizer introduces beneficial microbes that initiate and increase mutual plant–microbe interactions, stimulating roots to release root exudates, which support a wider range of microbial groups, ultimately resulting in a broader range of functions (Yusuf et al. 2025).

In contrast, chemical fertilizers deliver nutrients in a consistent and concentrated form, creating strong selection pressure that suppresses the growth of beneficial rhizosphere microbiomes (Enagbonma et al. 2024). This explains why there were relatively low functional categories in the chemically fertilized soil compared with those in the biofertilized soil. In general, biofertilizers promote a well‐rounded, adaptable, and robust microbial community. Chemical fertilizers, on the other hand, limit variety and decrease the overall stability of the ecosystem (Jana et al. 2024).

In the biofertilizer plot, the notably high relative abundance observed in our metagenome analysis was due to the prevalence of genes with unknown functions. Despite their functions being currently undefined, these genes may represent novel or poorly understood metabolic abilities within the microbial community. The prevalence of biofertilizers in biofertilizer‐treated soils suggests that biofertilizers may foster beneficial microbial communities with different and specialized functions not captured by recent annotation databases. Subsequent studies should focus on genes of unknown function and employ advanced metagenomics, recent annotation database transcriptomics, and functional techniques, which may reveal previously unrecognized biochemical processes and interactions that are essential for overall agroecosystem sustainability. The exploration of these uncharacterized genes presents valuable possibilities for broadening our knowledge of microbial contributions to soil quality and plant development.

Although this study focused on the fertilization response within a single season, microbial communities are likely to change over time with shifts in soil properties, organic matter dynamics, and microbial adaptation. However, to elucidate the long‐term effects of different fertilization regimes on soil microbial diversity and functions, we recommend that future studies incorporate multiseason trials to evaluate the long‐term effects of fertilization on soil microbial communities over time. Multiple‐season trials could reveal whether fertilization‐induced community changes are persistent, transient, or reversible and thus illuminate long‐term consequences for stress resistance, nutrient cycling efficiency, and specific functional pathways. Long‐term monitoring would also help assess microbial resilience and identify reliable soil health bioindicators, guiding sustainable management practices that protect microbial integrity while maintaining or improving crop yields.

We postulate that the difference in the microbial functional categories may have been affected by different fertilizer applications, impacting the recruitment of beneficial specific microbiomes and ultimately affecting the functional categories. Additional research is needed to elucidate the mechanisms that drive this particular microbial recruitment process.

## 5 Conclusion

In this study, we explored the categories of functions of the microbiomes of the *A. ampeloprasum* rhizosphere under chemical fertilizer and biofertilizer treatments, as well as in bulk soils via shotgun metagenomic sequencing. Our results indicate that all the types of fertilizer significantly affect microbial diversity and the functional categories present in the soil. The application of chemical fertilizers alters nutrient availability, which subsequently influences the microbial community and its functional characteristics. Importantly, compared with those fertilized with chemical options, soils treated with biofertilizers presented a broader range of functional categories, with bulk soils exhibiting even greater functional diversity. This investigation emphasized the distinct differences in functional categories between rhizosphere and bulk soils, which are largely influenced by the unique nutrient sources found in the rhizosphere.

Interestingly, in comparison with the plots treated with chemical fertilizers, the bulk soils presented a predominance of specific categories of functions, whereas the rhizosphere soils in those same plots presented reduced diversity. These findings highlight the necessity of managing fertilizer application to balance nutrient availability with biofertilizer use. To improve microbial functional diversity for sustainable agricultural practices, chemical fertilizers should be applied at moderate levels or be fully replaced with biofertilizers, which encourage a wider range of microbial functional categories. This shift can improve microbial functions essential for sustainable food production, thereby supporting SDG 2 (Zero hunger through improvements in soil fertility, crop yield, and overall agricultural productivity).

Additionally, this research indicates that the structure of the community of microorganisms and their functional diversities within the rhizosphere are maintained by both the establishment of the plant and the type of fertilizer used, with collaboration between the host plant and the rhizosphere microbiome being a key factor. A remarkable finding is the identification of an unknown functional category in the rhizosphere of plants treated with biofertilizers, implying that there are still uncharacterized microbial functions that could be sources of novel genes with considerable potential in agriculture and biotechnology. Further exploration of these less‐studied functional categories may open new possibilities for improving agricultural practices and sustainability.

## Author Contributions

**Oluwaseun Emmanuel Shittu:** methodology, software, data curation, investigation, formal analysis, visualization, writing – original draft, project administration. **Ben Jesuorsemwen Enagbonma:** methodology, software, data curation, supervision, writing – review and editing. **Olubukola Oluranti Babalola:** conceptualization, supervision, project administration, funding acquisition, investigation, validation, formal analysis, writing – review and editing.

## Ethics Statement

The authors have nothing to report.

## Consent

All authors gave their approval for the article's publication.

## Conflicts of Interest

The authors declare no conflicts of interest.

## Supporting information


**Figure S1:** Conceptual model of biofertilizer‐induced changes in rhizosphere microbiomes and plant functions.**Figure S2:** Heatmap depicting the relative abundance of microbial functional categories at EggNOG Level 1.**Figure S3:** Flowchart illustrating the metagenomic analysis pipeline, including the tools used at each step.**Figure S4:** Results of redundancy analysis (RDA) with forward selection of significant environmental variables affecting microbial functional categories.**Table S1:** Physicochemical properties of rhizosphere soil samples of Allium ampeloprasum rhizosphere (under chemical fertiliser and biofertilizer) and Bulk soil.**Table S2:** Analysis of sequenced data of the shotgun metagenome from the rhizosphere of the Allium ampeloprasum rhizosphere and uncultivated bulk soil.**Table S3:** Percentage (%) Abundance of Microbial Communities at the Order Level.**Table S4:** The relative abundance (percentage distribution) of the major functional categories in each soil sample at EggNOG level 1.**Table S5:** The relative abundance of the major functional categories in each soil sample at EggNOG level 2.**Table S6:** The abundance of the major pathways in each soil sample at KEGG level 3.**Table S7:** The abundance of the major functional categories in each soil sample with ANOVA analysis at EggNOG level 1.**Table S8:** Spearman s correlation coefficient.

## Data Availability

The data that support the high‐quality sequences of the findings of this study are openly available in the NCBI‐SRA data archive at http://identifiers.org/insdc.sra:SRP537120, http://identifiers.org/insdc.sra:SRP537121, and http://identifiers.org/insdc.sra:SRP537188 under the accession numbers SRP537120, SRP537121, and SRP537188, and the links are collectively deposited at https://doi.org/10.6084/m9.figshare.30285484.
