# Supplementary material for: Functional Metagenomics Insights Into the Allium ampeloprasum Rhizosphere Microbiome Under Different Fertilization Regimes
Source: Microbiologyopen. 2026 Jun 10;15(3):e70307. doi: 10.1002/mbo3.70307 (PMC13251443; doi:10.1002/mbo3.70307)
Supplement: Supplementary file 1 — Figure S1: Conceptual model of biofertilizer‐induced changes in rhizosphere microbiomes and plant functions.Figure S2: Heatmap depicting the relative abundance of microbial functional categories at EggNOG Level 1.Figure S3: Flowchart illustrating the metagenomic analysis pipeline, including the tools used at each step.Figure S4: Results of redundancy analysis (RDA) with forward selection of significant environmental variables affecting microbial functional categories.Table S1: Physicochemical properties of rhizosphere soil samples of Allium ampeloprasum rhizosphere (under chemical fertiliser and biofertilizer) and Bulk soil.Table S2: Analysis of sequenced data of the shotgun metagenome from the rhizosphere of the Allium ampeloprasum rhizosphere and uncultivated bulk soil.Table S3: Percentage (%) Abundance of Microbial Communities at the Order Level.Table S4: The relative abundance (percentage distribution) of the major functional categories in each soil sample at EggNOG level 1.Table S5: The relative abundance of the major functional categories in each soil sample at EggNOG level 2.Table S6: The abundance of the major pathways in each soil sample at KEGG level 3.Table S7: The abundance of the major functional categories in each soil sample with ANOVA analysis at EggNOG level 1.Table S8: Spearman s correlation coefficient. [file MBO3-15-e70307-s001.docx]

# Functional metagenomics insights into the *Allium ampeloprasum* rhizosphere microbiomes under different fertilization regimes

**Supplementary information**

**Table S1**: Physicochemical properties of rhizosphere soil samples of *Allium ampeloprasum* rhizosphere (under chemical fertiliser and biofertilizer) and Bulk soil

| Sample ID | L1 | L2 | L3 | L4 | L9 | L10 | L11 | L12 | LB1 | LB2 | LB3 | LB4 |
| --- | --- | --- | --- | --- | --- | --- | --- | --- | --- | --- | --- | --- |
| pH | 7.5 | 7.32 | 7.35 | 7.16 | 7.17 | 7.15 | 7.24 | 7.28 | 7.22 | 7.21 | 7.33 | 7.31 |
| Phosphorus (P) | 200 | 215 | 190 | 230 | 205 | 225 | 238 | 253 | 223 | 245 | 215 | 205 |
| Potassium (K) | 200 | 143 | 170 | 180 | 275 | 178 | 255 | 160 | 208 | 275 | 183 | 180 |
| Calcium (Ca) | 77 | 76.2 | 73.4 | 73 | 73 | 75.1 | 72.4 | 76.1 | 72.6 | 71.5 | 75.2 | 75 |
| Magnesium (Mg) | 15.4 | 18.1 | 19.2 | 19 | 16.5 | 17 | 17.5 | 16.6 | 18 | 17.9 | 17 | 17.1 |
| Sodium (Na) | 0 | 0 | 0.2 | 0.2 | 0 | 0.4 | 0 | 0.2 | 0.4 | 0 | 0 | 0 |
| Nitrate nitrogen (N-NO3) | 4.04 | 2.49 | 2.69 | 7.44 | 9.03 | 7.16 | 11.99 | 7.26 | 6.85 | 7.09 | 4.32 | 4.06 |
| Ammonium nitrogen (NNH4) | 1.45 | 1.05 | 1.3 | 1.5 | 2.05 | 1.55 | 1.75 | 1.55 | 1.15 | 1.2 | 1.5 | 1.85 |
| Total nitrogen (Total N) | 442 | 424 | 395 | 409 | 475 | 487 | 534 | 422 | 442 | 492 | 375 | 350 |
| Sand | 79 | 79 | 79 | 79 | 76 | 81 | 79 | 79 | 77 | 76 | 79 | 78 |
| silt | 11 | 9 | 6 | 7 | 8 | 8 | 9 | 8 | 7 | 8 | 7 | 9 |
| Clay | 10 | 12 | 15 | 14 | 16 | 11 | 12 | 13 | 16 | 16 | 14 | 13 |
| Carbon (C) | 0.55 | 0.48 | 0.55 | 0.53 | 0.59 | 0.66 | 0.68 | 0.5 | 0.52 | 0.63 | 0.5 | 0.43 |
| S-Value | 6.711 | 6.513 | 6.031 | 5.88 | 6.746 | 6.061 | 6.509 | 5.739 | 5.878 | 6.678 | 6.067 | 5.803 |
| Moisture content | 3.7 | 4.9 | 6.6 | 9.9 | 8.7 | 4.8 | 4.2 | 6.6 | 6.2 | 4.6 | 4.1 | 5.7 |

**Table S2:** Analysis of sequenced data of the shotgun metagenome from the rhizosphere of the *Allium ampeloprasum* rhizosphere and uncultivated bulk soil

| Sample ID | Total len (bp) | Scaftigs num | Average len(bp) | N50 len(bp) | N90 len(bp) | Max len(bp) |
| --- | --- | --- | --- | --- | --- | --- |
| L1 | 207 133 575 | 279 989 | 739.79 | 696 | 526 | 37 428 |
| L2 | 220 163 088 | 293 663 | 749.71 | 708 | 527 | 18 728 |
| L3 | 230 004 647 | 305 830 | 752.07 | 709 | 528 | 29 278 |
| L4 | 189 398 882 | 254 560 | 744.02 | 697 | 525 | 16 528 |
| L9 | 285 268 861 | 316 496 | 901.33 | 794 | 533 | 1 006 233 |
| L10 | 163 046 484 | 223 983 | 727.94 | 674 | 523 | 46 653 |
| L11 | 224 304 831 | 310 609 | 722.15 | 678 | 524 | 29 791 |
| L12 | 190 484 290 | 254 888 | 747.33 | 695 | 525 | 52 627 |
| LB1 | 205 215 937 | 279 183 | 735.06 | 699 | 527 | 9 711 |
| LB2 | 289 466 119 | 384 519 | 752.8 | 716 | 529 | 24 859 |
| LB3 | 148 202 624 | 207 600 | 713.89 | 672 | 523 | 10 154 |
| LB4 | 166 948 562 | 228 005 | 732.21 | 689 | 525 | 15 500 |

Three groups, G1, G2, and G3, each consisting of 4 replicates. G1 (Soil samples from chemical fertiliser plot) = L1, L2, L3 and L4; G2(Soil samples from biofertiliser plot) = (L9, L10, L11 and L12); and G3(Soil samples from uncultivated bulk soils) = (LB1, LB2, LB3 and LB4).

**Table S3**: Percentage (%) Abundance of Microbial Communities at the Order Level

| Order | G1 | G2 | G3 |
| --- | --- | --- | --- |
| Propionibacteriales | 13.28 ± 2.284a | 14.15 ± 2.401a | 14.90 ± 0.587a |
| Micrococcales | 6.49 ± 1.029a | 6.48 ± 0.451a | 4.90 ± 0.233a |
| Cytophagales | 0.09 ± 0.021a | 1.92 ± 1.836a | 0.09 ± 0.011a |
| Hyphomicrobiales | 4.62 ± 0.317a | 5.52 ± 0.190a | 5.21 ± 0.763a |
| Verrucomicrobiales | 0.05 ± 0.009a | 1.62 ± 1.583a | 0.03 ± 0.002a |
| Solirubrobacterales | 4.19 ± 0.364a | 3.91 ± 0.578a | 4.65 ± 0.492a |
| Polyangiales | 0.08 ± 0.011a | 1.06 ± 0.973a | 0.08 ± 0.011a |
| Kitasatosporales | 0.93 ± 0.147a | 2.30 ± 0.847a | 0.73 ± 0.043a |
| Rubrobacterales | 2.06 ± 0.571a | 1.08 ± 0.159a | 1.93 ± 0.489a |
| Haliangiales | 0.02 ± 0.005a | 0.93 ± 0.916a | 0.02 ± 0.004a |
| Burkholderiales | 0.79 ± 0.009a | 1.55 ± 0.671a | 0.67 ± 0.039a |
| Xanthomonadales | 0.34 ± 0.046a | 0.97 ± 0.646a | 0.24 ± 0.028a |
| Pseudomonadales | 0.74 ± 0.638a | 0.24 ± 0.110a | 0.07 ± 0.018a |
| Gaiellales | 1.79 ± 0.192a | 1.85 ± 0.211a | 2.05 ± 0.204a |
| Sphingomonadales | 1.64 ± 0.079a | 1.67 ± 0.220a | 1.59 ± 0.044a |
| Nitrososphaerales | 1.17 ± 0.117a | 1.20 ± 0.074a | 1.47 ± 0.137a |
| Myxococcales | 0.31 ± 0.020a | 0.87 ± 0.393a | 0.35 ± 0.042a |
| Mycobacteriales | 0.74 ± 0.357a | 0.46 ± 0.038a | 0.46 ± 0.031a |
| Bacillales | 0.57 ± 0.057a | 0.80 ± 0.134a | 0.97 ± 0.220a |
| Nitrospirales | 1.15 ± 0.155a | 0.95 ± 0.127a | 1.13 ± 0.118a |
| Geodermatophilales | 0.93 ± 0.181a | 1.05 ± 0.219a | 0.96 ± 0.091a |
| Thermomicrobiales | 1.12 ± 0.061a | 0.93 ± 0.116a | 1.16 ± 0.090a |
| Rhodobacterales | 0.57 ± 0.233a | 0.54 ± 0.121a | 0.22 ± 0.052a |
| Acidimicrobiales | 0.81 ± 0.069a | 0.75 ± 0.104a | 0.94 ± 0.045a |
| Gemmatales | 0.53 ± 0.167a | 0.24 ± 0.019a | 0.31 ± 0.029a |
| Gemmatimonadales | 0.80 ± 0.028a | 0.67 ± 0.062b | 0.82 ± 0.017a |
| Pseudonocardiales | 0.61 ± 0.068a | 0.61 ± 0.099a | 0.65 ± 0.044a |
| Rhodospirillales | 0.50 ± 0.040a | 0.59 ± 0.020a | 0.54 ± 0.039a |
| Chitinophagales | 0.09 ± 0.015a | 0.20 ± 0.127a | 0.08 ± 0.009a |
| Longimicrobiales | 0.22 ± 0.120a | 0.14 ± 0.035a | 0.14 ± 0.020a |
| Anaerolineales | 0.40 ± 0.037a | 0.41 ± 0.070a | 0.43 ± 0.055a |
| Micromonosporales | 0.36 ± 0.025a | 0.33 ± 0.047a | 0.36 ± 0.022a |
| Nitrosopumilales | 0.23 ± 0.061a | 0.28 ± 0.034a | 0.35 ± 0.042a |
| Vicinamibacterales | 0.31 ± 0.030a | 0.21 ± 0.012b | 0.27 ± 0.008ab |
| Pirellulales | 0.29 ± 0.041a | 0.26 ± 0.047a | 0.21 ± 0.017a |

G1 (Soil samples from chemical fertilizer plot)

G2(Soil samples from biofertilizer plot)

G3(Soil samples from uncultivated bulk soils)

**Table S4**: The relative abundance (percentage distribution) of the major functional categories in each soil sample at EggNOG level 1

| Functional Category | G1 | G2 | G3 |
| --- | --- | --- | --- |
| Function unknown | 13,36 | 13,93 | 13,32 |
| Amino acid transport and metabolism | 8,57 | 8,41 | 8,61 |
| Energy production and conversion | 7,02 | 6,87 | 7,02 |
| Carbohydrate transport and metabolism | 5,66 | 5,73 | 5,61 |
| Transcription | 5,45 | 5,70 | 5,45 |
| Replication, recombination and repair | 5,14 | 5,21 | 5,17 |
| Cell wall/membrane/envelope biogenesis | 4,81 | 4,87 | 4,79 |
| Signal transduction mechanisms | 4,68 | 4,81 | 4,66 |
| Inorganic ion transport and metabolism | 4,95 | 4,96 | 4,92 |
| Translation, ribosomal structure and biogenesis | 4,67 | 4,57 | 4,68 |
| Lipid transport and metabolism | 3,90 | 3,91 | 3,92 |
| Coenzyme transport and metabolism | 3,73 | 3,65 | 3,75 |
| Posttranslational modification, protein turnover, chaperones | 3,35 | 3,33 | 3,37 |
| Secondary metabolites biosynthesis, transport and catabolism | 2,87 | 2,89 | 2,85 |
| Nucleotide transport and metabolism | 2,56 | 2,52 | 2,58 |
| Intracellular trafficking, secretion, and vesicular transport | 1,95 | 1,95 | 1,90 |
| Defense mechanisms | 1,83 | 1,79 | 1,82 |
| Cell cycle control, cell division, chromosome partitioning | 1,48 | 1,51 | 1,50 |
| Cell motility | 0,93 | 0,99 | 0,91 |
| Cytoskeleton | 0,17 | 0,19 | 0,17 |
| RNA processing and modification | 0,05 | 0,05 | 0,05 |
| Extracellular structures | 0,02 | 0,02 | 0,02 |
| Chromatin structure and dynamics | 0,04 | 0,04 | 0,04 |

**Table S5**: The relative abundance of the major functional categories in each soil sample at EggNOG level 2

| Description | G1 | G2 | G3 |
| --- | --- | --- | --- |
| Transcriptional regulator | 53.69 | 58.79 | 52.27 |
| Phosphorelay signal transduction system | 55.18 | 56.63 | 55.38 |
| ATPase activity | 54.58 | 54.34 | 54.99 |
| Histidine kinase | 44.05 | 45.95 | 44.20 |
| ABC transporter | 47.30 | 47.71 | 47.47 |
| protein histidine kinase activity | 39.94 | 40.78 | 40.11 |
| Protein conserved in bacteria | 35.37 | 36.64 | 34.40 |
| (ABC) transporter | 36.30 | 36.36 | 36.38 |
| Major facilitator Superfamily | 34.41 | 34.66 | 34.46 |
| rRNA binding | 32.07 | 31.13 | 31.91 |
| transferase activity, transferring glycosyl groups | 30.16 | 29.67 | 30.42 |
| protein kinase activity | 17.59 | 19.52 | 16.81 |
| Serine threonine protein kinase | 17.49 | 19.48 | 16.82 |
| DNA-binding transcription factor activity | 22.84 | 25.35 | 21.71 |
| oxidoreductase activity, acting on CH-OH group of donors | 25.27 | 26.58 | 25.29 |
| transcriptional regulator | 23.73 | 26.12 | 22.53 |
| response regulator | 26.75 | 26.31 | 26.77 |
| sigma factor activity | 22.97 | 23.85 | 23.34 |
| Major Facilitator Superfamily | 22.85 | 23.02 | 22.94 |
| Tetratricopeptide repeat | 17.04 | 17.83 | 16.48 |
| Transport | 16.10 | 17.10 | 15.40 |
| acyl-CoA dehydrogenase activity | 20.64 | 20.48 | 21.36 |
| sequence-specific DNA binding | 12.52 | 15.99 | 12.32 |
| electron transfer activity | 15.94 | 16.61 | 15.13 |
| peptidyl-tyrosine sulfation | 15.91 | 16.53 | 15.30 |
| DNA-directed 5_-3_ RNA polymerase activity | 20.17 | 19.75 | 21.10 |
| Short-chain dehydrogenase reductase Sdr | 18.74 | 19.69 | 18.68 |
| DNA-dependent RNA polymerase catalyzes the transcription of (...) | 19.41 | 18.97 | 20.33 |
| acyl-CoA dehydrogenase | 17.85 | 17.98 | 18.57 |
| efflux transmembrane transporter activity | 16.74 | 14.45 | 15.76 |
| RESPONSE REGULATOR receiver | 17.12 | 17.62 | 16.90 |
| metalloendopeptidase activity | 19.29 | 18.71 | 19.88 |
| ligase activity | 18.70 | 18.36 | 18.89 |
| Binding-protein-dependent transport system inner membrane co(...) | 18.24 | 18.77 | 18.50 |
| carbohydrate transport | 17.29 | 18.06 | 16.94 |

**Table S6**: The abundance of the major pathways in each soil sample at KEGG level 3

|  | KEGG Orthologous code | Pathway | G1 | G2 | G3 |
| --- | --- | --- | --- | --- | --- |
| Amino acid pathway | ko00220 | Arginine biosynthesis | 49.13 | 48.45 | 50.00 |
|  | ko00260 | Glycine, serine and threonine metabolism | 80.63 | 77.60 | 79.90 |
|  | ko00280 | Valine, leucine and isoleucine degradation | 83.37 | 81.34 | 83.40 |
|  | ko00310 | lysine degradation | 41.67 | 41.60 | 41.83 |
|  | ko00380 | Tryptophan metabolism | 45.88 | 44.73 | 45.78 |
|  | ko00330 | Arginine and proline metabolism | 45.63 | 43.28 | 45.65 |
| Carbohydrate pathway | ko00010 | Regulation of glycolysis/gluconeogenesis | 96.63 | 94.09 | 97.26 |
|  | ko00020 | Citrate cycle (TCA cycle) | 94.09 | 91.92 | 94.27 |
|  | ko00030 | The pentose phosphate pathway | 52.72 | 52.56 | 53.44 |
|  | ko00500 | Starch and sucrose metabolism | 63.41 | 62.66 | 63.33 |
|  | ko00630 | Glutamine synthetase-glutamate synthase pathway | 126.06 | 122.66 | 127.42 |
|  | ko00310 | lysine degradation | 41.67 | 41.60 | 41.83 |
|  | ko00640 | Propanoate metabolism | 74.68 | 72.10 | 74.25 |
| Lipid Pathway | ko00640 | Propanoate metabolism | 74.68 | 72.10 | 74.25 |
|  | ko00520 | Amino sugar and nucleotide sugar metabolism | 69.30 | 67.91 | 69.07 |
| Inorganic ions Pathway | ko00910 | Nitrogen metabolism | 51.38 | 50.85 | 51.26 |
|  | ko02020 | Two-component signal transduction systems | 134.43 | 137.13 | 132.91 |
|  | ko02010 | ATP-binding cassette (ABC) transporter substrate-binding protein | 191.24 | 191.89 | 189.77 |
|  | ko00970 | Aminoacyl-tRNA biosynthesis | 92.92 | 89.93 | 94.01 |
|  | ko00770 | Pantothenate and CoA biosynthesis | 43.72 | 41.70 | 43.77 |
| Phosphorus Pathway | ko02020 | Two-component signal transduction systems (Phosphate Transport and uptake) | 134.43 | 137.13 | 132.91 |
|  | ko00640 | Propanoate metabolism | 74.68 | 72.10 | 74.25 |
| Secondary metabolites Pathway | ko00310 | Volatile Organic Compounds (VOCs) Biosynthesis | 41.67 | 41.60 | 41.83 |
|  | ko00250 | Ethylene Biosynthesis | 97.73 | 95.63 | 99.28 |
| Nitrogen metabolism pathway | ko00910 | Nitrogen metabolism | 51.38 | 50.85 | 51.26 |

**Table S7**: The abundance of the major functional categories in each soil sample with ANOVA analysis at EggNOG level 1

| Functions | G1 | G2 | G3 |
| --- | --- | --- | --- |
| Amino acid transport and metabolism | 8.57 ± 0.103a | 8.41 ± 0.335a | 8.61± 0.02a |
| Carbohydrate transport and metabolism | 5.66 ± 0.125a | 5.73 ± 0.071a | 5.61± 0.042a |
| Cell cycle control, cell division, chromosome partitioning | 1.48 ± 0.042a | 1.51 ± 0.025a | 1.50± 0.018a |
| Cell motility | 0.93 ± 0.015a | 0.99 ± 0.061a | 0.91± 0.017a |
| Cell wall/membrane/envelope biogenesis | 4.81 ± 0.026a | 4.87 ± 0.119a | 4.79± 0.012a |
| Chromatin structure and dynamics | 0.04 ± 0a | 0.04 ± 0.002a | 0.04± 0.001a |
| Coenzyme transport and metabolism | 3.73 ± 0.014a | 3.65 ± 0.096a | 3.75± 0.008a |
| Cytoskeleton | 0.17 ± 0.004a | 0.19 ± 0.023a | 0.17± 0.001a |
| Defense mechanisms | 1.83 ± 0.01a | 1.79 ± 0.028a | 1.82± 0.01a |
| Energy production and conversion | 7.02 ± 0.065a | 6.87± 0.26a | 7.02± 0.027a |
| Extracellular structures | 0.02 ± 0.002a | 0.02± 0.009a | 0.02± 0.001a |
| Function unknown | 13.36 ± 0.167a | 13.93± 0.345a | 13.3± 0.034a |
| Inorganic ion transport and metabolism | 4.95 ± 0.061a | 4.96± 0.044a | 4.92± 0.017a |
| Intracellular trafficking, secretion, and vesicular transport | 1.95 ± 0.027a | 1.95± 0.07a | 1.90± 0.019a |
| Lipid transport and metabolism | 3.9 ± 0.101a | 3.91± 0.117a | 3.92± 0.024a |
| Nucleotide transport and metabolism | 2.56 ± 0.04a | 2.52± 0.1a | 2.58± 0.017a |
| Posttranslational modification, protein turnover, chaperones | 3.35 ± 0.011a | 3.33± 0.034a | 3.37± 0.011a |
| RNA processing and modification | 0.05 ± 0.001a | 0.05± 0.005a | 0.05± 0.001a |
| Replication, recombination and repair | 5.14 ± 0.111a | 5.21± 0.115a | 5.17± 0.039a |
| Secondary metabolites biosynthesis, transport and catabolism | 2.87 ± 0.06a | 2.89± 0.052a | 2.85± 0.014a |
| Signal transduction mechanisms | 4.68 ± 0.027a | 4.81± 0.133a | 4.66± 0.004a |
| Transcription | 5.45 ± 0.137a | 5.7 0± 0.093a | 5.45± 0.047a |
| Translation, ribosomal structure and biogenesis | 4.67 ± 0.077a | 4.57± 0.184a | 4.68± 0.029a |

**Table S8:** Spearman’s correlation coefficient

|  | pH | Phosphorus (P) | Potassium (K) | Calcium (Ca) | Magnesium (Mg) | Sodium (Na) | Nitrate nitrogen (N-NO3) | Ammonium nitrogen (NNH4) | Total nitrogen (Total N) | Sand | silt | Clay | Carbon (C) | S-Value | Moisture content |
| --- | --- | --- | --- | --- | --- | --- | --- | --- | --- | --- | --- | --- | --- | --- | --- |
| Function unknown | -0.54545 | 0.329827 | 0.26316 | 0.010508 | -0.63047 | 0.216815 | 0.643357 | 0.463161 | 0.290718 | -0.02735 | -0.06502 | 0.070802 | 0.333335 | 0.111888 | 0.234676 |
| Amino acid | -0.13287 | 0.333335 | -0.04561 | 0.182137 | -0.30473 | 0.315367 | 0.293706 | 0.189475 | 0.206655 | 0.644738 | 0.191437 | -0.64076 | 0.312283 | -0.1958 | -0.4028 |
| Energy | 0.097902 | 0.280703 | 0.021053 | 0.241682 | -0.43082 | -0.01971 | 0.216783 | 0.284212 | 0.161121 | 0.558773 | 0.408158 | -0.71156 | 0.189475 | -0.11888 | -0.63748 |
| Carbohydrate | -0.30769 | 0.400002 | 0.214036 | -0.00701 | -0.35026 | 0.295656 | 0.461538 | 0.126317 | 0.462347 | 0.402473 | 0.130033 | -0.40003 | 0.561407 | 0.06993 | -0.2697 |
| Transcription | -0.62238 | 0.312283 | 0.29123 | -0.04553 | -0.58844 | 0.295656 | 0.643357 | 0.431582 | 0.364274 | -0.03517 | -0.06502 | 0.070802 | 0.396494 | 0.118881 | 0.245184 |
| Replication | -0.06294 | 0.438599 | 0.031579 | 0.203153 | -0.44834 | 0.236525 | 0.13986 | -0.01053 | 0.304729 | 0.42201 | 0.166153 | -0.44605 | 0.308774 | -0.07692 | -0.56392 |
| Cell wall | -0.1958 | -0.13333 | -0.00702 | -0.28371 | 0.409808 | 0.177394 | 0.090909 | -0.08772 | -0.28021 | -0.49625 | -0.4804 | 0.690318 | -0.30176 | -0.16084 | 0.861648 |
| Signal transduction | -0.28671 | 0.280703 | 0.014035 | 0.105079 | -0.21366 | -0.13797 | 0.27972 | 0.098246 | -0.14011 | -0.34777 | -0.08308 | 0.343389 | -0.38597 | 0.041958 | 0.455342 |
| Inorganic ion transport and metabolism | 0 | 0.315791 | 0.02807 | 0.157618 | -0.29422 | 0.059131 | 0.293706 | 0.23158 | 0.140105 | 0.54705 | 0.307021 | -0.63722 | 0.266668 | -0.11888 | -0.43082 |
| Translation | 0.055944 | 0.26316 | 0.024562 | 0.280211 | -0.42382 | 0.177394 | 0.160839 | 0.084211 | 0.161121 | 0.59394 | 0.155317 | -0.58058 | 0.256142 | -0.06993 | -0.59895 |
| Lipid transport | -0.12587 | 0.410529 | -0.05965 | 0.262697 | -0.33625 | 0.236525 | 0.244755 | 0.059649 | 0.19965 | 0.582218 | 0.220333 | -0.60536 | 0.245616 | -0.1049 | -0.3958 |
| Coenzyme | 0.34965 | -0.17193 | 0.045614 | 0.052539 | -0.10158 | -0.23652 | -0.23077 | 0.189475 | -0.08757 | 0.269618 | 0.292573 | -0.43189 | 0.056141 | -0.0979 | -0.69352 |
| Posttranslational | 0.153846 | 0.238598 | 0.168422 | 0.171629 | -0.32224 | 0.118262 | 0.06993 | -0.10877 | 0.094571 | 0.34386 | 0.05418 | -0.34339 | 0.056141 | -0.0979 | -0.63398 |
| Secondary metabolites | -0.12587 | 0.343862 | -0.17544 | 0.353766 | -0.36427 | 0.236525 | 0.195804 | 0.178948 | 0.031524 | 0.64083 | 0.205885 | -0.64784 | 0.119299 | -0.20979 | -0.36077 |
| Nucleotide | -0.08392 | 0.305265 | 0.105264 | 0.136603 | -0.31524 | 0.137973 | 0.195804 | 0.063158 | 0.238179 | 0.56268 | 0.115584 | -0.52039 | 0.37895 | 0.027972 | -0.61997 |
| Intracellular | 0.167832 | -0.38246 | -0.29123 | 0.115587 | 0.255692 | -0.21681 | -0.0979 | 0.143861 | -0.47286 | -0.17193 | -0.1517 | 0.261967 | -0.42807 | -0.01399 | 0.640982 |

**Table S8: Composition, dosage, and application details of chemical and biofertilizer used**

| **S/N** | **Treatment type** | **Specific name** | **Key composition** | **Microbial load (CFU g⁻¹)** | **Dosage or rate (mL/l/g/m^2^)** | **Timing and method of application** |
| --- | --- | --- | --- | --- | --- | --- |
| 1. | Chemical fertilizers used in plot A (G1) | Calcium nitrate (Ca(NO_3_)_2_) | 15.5% total nitrogen, and 19% of calcium | - | 150g | Applied at the pre‑planting and vegetative growth stages via soil incorporation as a basal application and by foliar spraying. |
|  |  | Ammonium sulphate ((NH_4_)_2_SO_4_) | 21% of nitrogen and 24% of sulfur | - | 120g | Applied during the vegetative growth stage as a side‑dressing treatment around the base of the plants. |
|  |  | Potassium nitrate (KNO_3_) | 13% total nitrogen and 46% of potassium | - | 200g | Applied during the vegetative growth stage via foliar application. |
|  |  | Magnesium nitrate (Mg(NO_3_)_2_) | 11% nitrogen (N) in nitrate form, 16% magnesium oxide (MgO), and 9.6% magnesium (Mg) | - | 150g | Applied at the pre-planting stage via a soil drench application. |
|  |  | Potassium sulphate (K_2_SO_4_) | 53% potassium, and 18% sulfur | - | 150g | Applied at the bulbing stage through incorporation into the soil. |
|  |  | N: P: K (5:1:5) | 5parts Nitrogen, 1part potassium, and 5parts potassium | - | 100g | Applied as a pre-plant basal application using broadcast distribution. |
| 2. | Biofertilizers used in plot B (G2) | Terramax | A natural organic extract comprising a variety of plant growth-enhancing rhizobacteria (PGPRs) | - | 0.56g | Applied at the pre-planting phase and during the vegetative growth stage through soil drenching. |
|  |  | Humesoil | an organic formulation derived from plants and trees | - | 0.82 litre | Applied prior to planting by means of soil drenching. |
|  |  | Soluphos | A Phosphate-solubilizing microbial inoculant in organic powder form that contains *Pseudomonas putida*, *Bacillus licheniformis* | 4.8 × 10^9^ | 0.494 ml | Applied through fertigation at pre-planting and during the early vegetative growth stage |


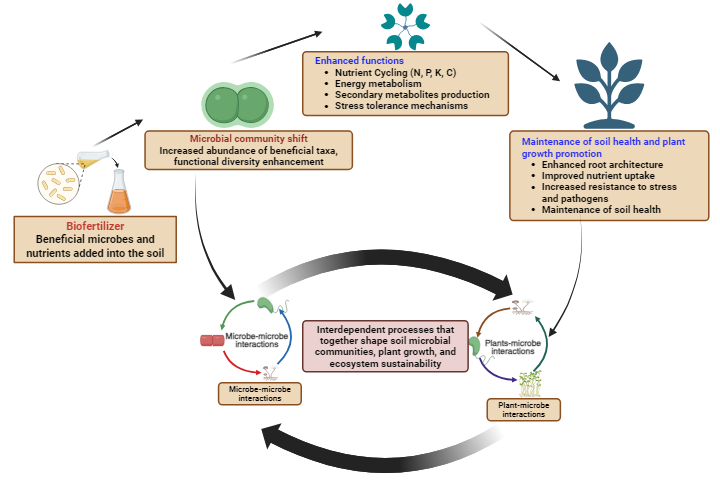


**Figure S1.** Conceptual model of biofertilizer-induced changes in rhizosphere microbiomes and plant functions.


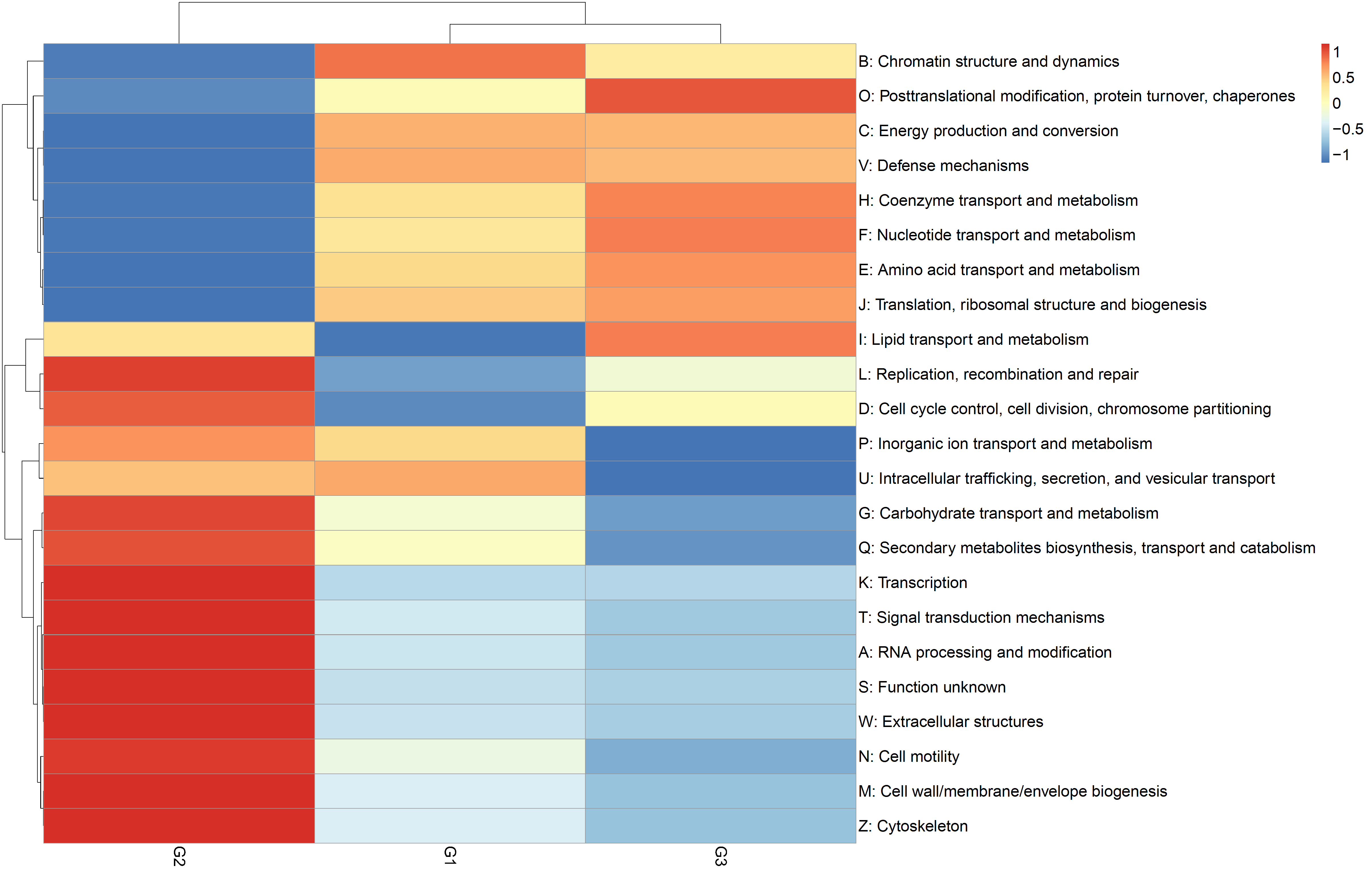


**Figure S2:** Heatmap depicting the relative abundance of microbial functional categories at EggNOG Level 1.


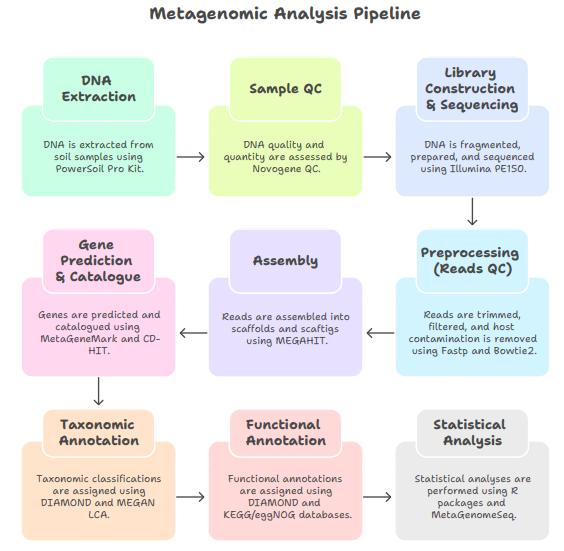


**Figure S3:** Flowchart illustrating the metagenomic analysis pipeline, including the tools used at each step.

**Figure S4:** Results of redundancy analysis (RDA) with forward selection of significant environmental variables affecting microbial functional categories
